# Experimental validation of FINDSITE^comb^ virtual ligand screening results for eight proteins yields novel nanomolar and micromolar binders

**DOI:** 10.1186/1758-2946-6-16

**Published:** 2014-04-26

**Authors:** Bharath Srinivasan, Hongyi Zhou, Julia Kubanek, Jeffrey Skolnick

**Affiliations:** 1 Center for the Study of Systems Biology, School of Biology, Georgia Institute of Technology, 250, 14th Street, N.W., Atlanta, GA 30318, USA; 2 School of Biology, Atlanta, GA 30332, USA; 3 School of Chemistry and Biochemistry, Aquatic Chemical Ecology Center, Institute of Bioengineering and Biosciences, Georgia Institute of Technology, Atlanta, GA 30332, USA

**Keywords:** Drug discovery, Virtual ligand screening (VLS), High-throughput screening (HTS), Differential scanning fluorimetry (DSF), Ligand homology modeling

## Abstract

**Background:**

Identification of ligand-protein binding interactions is a critical step in drug discovery. Experimental screening of large chemical libraries, in spite of their specific role and importance in drug discovery, suffer from the disadvantages of being random, time-consuming and expensive. To accelerate the process, traditional structure- or ligand-based VLS approaches are combined with experimental high-throughput screening, HTS. Often a single protein or, at most, a protein family is considered. Large scale VLS benchmarking across diverse protein families is rarely done, and the reported success rate is very low. Here, we demonstrate the experimental HTS validation of a novel VLS approach, FINDSITE^comb^, across a diverse set of medically-relevant proteins.

**Results:**

For eight different proteins belonging to different fold-classes and from diverse organisms, the top 1% of FINDSITE^comb^’s VLS predictions were tested, and depending on the protein target, 4%-47% of the predicted ligands were shown to bind with μM or better affinities. In total, 47 small molecule binders were identified. Low nanomolar (nM) binders for dihydrofolate reductase and protein tyrosine phosphatases (PTPs) and micromolar binders for the other proteins were identified. Six novel molecules had cytotoxic activity (<10 μg/ml) against the HCT-116 colon carcinoma cell line and one novel molecule had potent antibacterial activity.

**Conclusions:**

We show that FINDSITE^comb^ is a promising new VLS approach that can assist drug discovery.

## Background

Traditional experimental approaches to drug discovery rely on two different strategies [1]. The first selects a reliable therapeutic target that might be essential for an organism’s or cell’s survival, and then, using chemical library screening, potential leads that bind to and modulate the activity of the target *in vitro* and subsequently, *in vivo*, are identified. The second approach tests small molecules on animal disease models or cell cultures (called phenotypic screening), and once activity is gleaned, the protein target is experimentally identified by target deconvolution [2]. Both approaches have contributed to the discovery of new drugs despite suffering from substantial disadvantages of high cost and time. Fragment-based drug discovery approaches have recently gained prominence as a distinct and complementary approach to drug discovery [3]. Integration of a robust VLS methodology with experimental HTS approaches constitutes one of the many methods that might accelerate the drug discovery process [4].

Despite its current limitations, VLS may be employed as a possible first step in drug discovery [5]. It not only aids in the selection of an appropriate protein target but also narrows down the chemical space that is experimentally screened to arrive at significant protein-ligand interactions. In practice, both ligand- and structure-based VLS approaches [6] have been used. The principal disadvantage of a ligand-based approach is the need for *a priori* knowledge of a set of ligands known to bind to the target [7]. Structure-based approaches require a high-resolution structure of the target; this situation typically only holds for a minority of proteins in a given proteome [8]. To overcome these limitations, ligand homology modeling (LHM) was developed to predict ligands that bind to the protein target [9-11]. LHM relies on the fact that evolutionarily distant proteins share functional overlap and their ligand-binding information provides diverse bound ligands that can be employed in a general VLS approach. Thus, it does not suffer from the limitations of quantitative structure-activity relationship (QSAR)-based approaches. In large scale benchmarking, the FINDSITE^comb^ LHM approach exhibited significant performance advantages over traditional approaches in terms of enrichment factor, speed, and insensitivity as to whether experimental or predicted protein structures are used [12]. However, experimental assessment of the method, where blind predictions are made and then experimentally tested, has not been done. To ensure robustness, a diverse set of proteins and ligands must be examined, and the strengths and limitations of the approach demonstrated.

A reliable and fast method that would test VLS predictions and identify hits could help accelerate the drug-discovery process. This could help alleviate the inherent complexity of treating diseases due to cross-reactivity and could address the rapid evolution of resistance to available drugs by pathogenic microbes. We have resorted to the thermal shift assay methodology to assess the predictions from VLS [13]. The methodology is an inexpensive way to assess the binding of small-molecules by the stability they confer on thermal denaturation of the protein target of interest. Upon thermal denaturation, the hydrophobicity of proteins increases, leading to an increase in fluorescence of an extrinsic fluorophore reporter dye. This method is amenable to miniaturization and can screen hundreds of molecules simultaneously for their ability to bind to the protein target of interest.

Recognizing the importance of these issues, in the present paper, to assess if FINDSITE^comb ^[12] can improve VLS, we selected an assortment of medically-relevant proteins with differing fold-architectures from diverse organisms including the causative agents of human and primate malaria, *Plasmodium falciparum* and *Plasmodium knowlesi*, an opportunistic pathogen *Escherichia coli*, and proteins implicated in mammalian disorders (from *Homo sapiens* and *Rattus norvegicus*). For these proteins, top ranked ligands predicted by FINDSITE^comb^ are experimentally assessed for binding by thermal-melt assays. After validating the small molecule binding predictions, we tested their physiological function by their ability to kill bacteria such as multi-drug resistant *E. coli* (MDREC), methicillin-resistant *Staphylococcus aureus* (MRSA), Vancomycin-resistant *Enterococcus faecium* (VREF), and their cytotoxic activity using HCT-116 colon carcinoma tumor cell line. The encouraging experimental results for both binding and physiological activity show that FINDSITE^comb^ is an effective VLS tool.

## Results

The section summarizes the results from FINDSITE^comb^’s VLS predictions on eight different proteins and their validation by the thermal shift assay methodology.

Prior to assessing the VLS results on the eight protein test set, the thermal shift methodology was validated on three proteins having known binding and nonbinding ligands. Only cognate protein-ligand pairs showed shifts in the transition mid-point of thermal melt curves, *T*_m_, while non-cognate ligands displayed no such shifts (Additional file 1: Figure S1 and SI).

**Figure 1:**
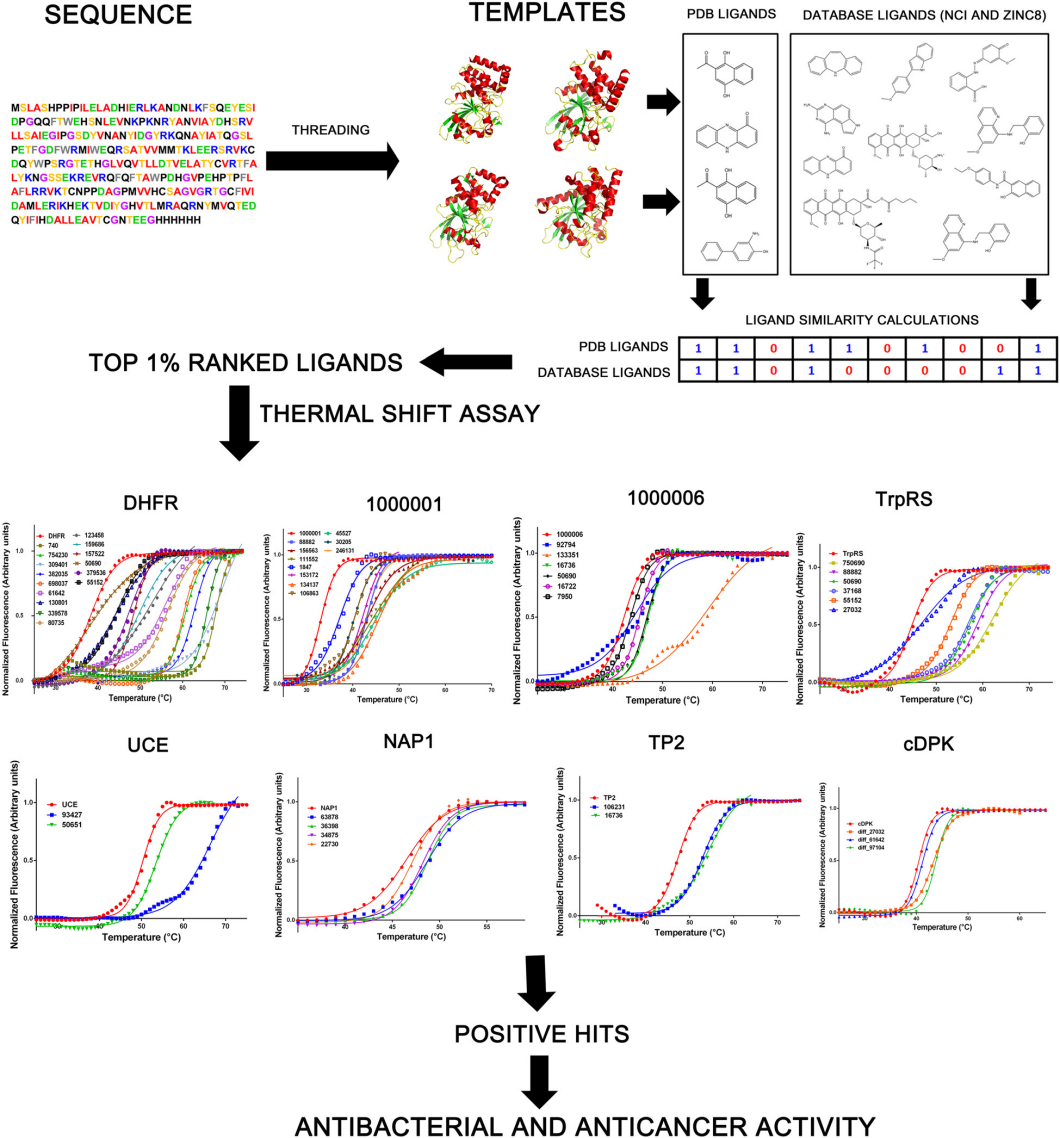
**Flowchart of the overall approach and the thermal shift assay results.** The first panel shows the *in silico* approach to predicting protein-small molecule interactions. All predictions were in benchmarking mode with a 30% template SID cutoff and the top 1% of the hits tested using thermal-shift assays. The second panel shows a representative fraction of the thermal melt curves that showed positive shifts for the tested proteins. The numbers are the NSC notation that identifies each small-molecule. DHFR is *E. coli* dihydrofolate reductase, 1000001 is a PTP from *R. norvegicus*, 1000006 is a PTP from *H. sapiens*, TrpRS is tryptophanyl tRNA synthetase from *H. sapiens*, UCE is ubiquitin-conjugating enzyme from *P. falciparum*, NAP1 is nucleosome assembly protein 1 from *P. knowlesi*, TP2 is thioredoxin peroxidase 2 from *P. falciparum* and cDPK is the wild-type cAMP-dependent protein kinase, catalytic subunit from *H. sapiens*. Small-molecule binders were tested for their antimicrobial & cytotoxic activity against HCT-116 colon carcinoma cell line.

We next applied the methodology, as shown in Figure 1, in benchmark mode to eight diverse proteins, viz., FINDSITE^comb^ only considered template proteins whose sequence identities to the target was <30%. Typically on the order of 50 ligands per protein gave interpretable thermal shift curves. Of these, the experiments identified a total of 47 small-molecule/protein binding interactions with μM or better affinities. Ten ligands with apparent nM binding affinities (less than 1 μM) were identified for dihydrofolate reductase from *E. coli* and the two mammalian protein tyrosine phosphatases (PTPs). Except for a small fraction of known inhibitors, which further validated the methodology, most are novel. The prediction percentage success rate ranged from 3.9% of ligands tested for the *P. falciparum* ubiquitin-conjugating enzyme to almost 47% for dihydrofolate reductase from *E. coli* (Table 1). This is a major advancement over previously reported success rates [14]. The small-molecules that displayed biological activity had low μM or nM affinities in the *in vitro* thermal shift assay (Table 2; Additional file 1: Tables S3-S5). This supports the conjecture that their *in vivo* biological activity might result from binding of the small-molecule with the intended target protein. A more detailed summary of the results is presented below.

**Table 1:** Results from the thermal shift assays on eight proteins, ranked by best ligand binding*

**Protein**	**Organism**	**No. of ligands tested**	**No. of good curves**	**No. of + ve**^**a **^**shifts/% + ve**^**a **^**shifts**	**Best hit (NSC)**	**Δ** ***T***_**m **_**(°C)**	***K***_**D **_**(nM)**^**b**^	**Best hit structure**
DHFR	*E. coli*	83	32	15/46.9	309401	30.74	48.21	
1000006	*H. sapiens*	59	43	6/13.9	133351	16.76	168.29	
1000001	*R. norvegicus*	86	42	10/23.8	134137	12.30	406.0	
TrpRS	*H. sapiens*	94	12	5/41.7	750690	14.57	1277.51	
UCE	*P. falciparum*	80	51	2/03.9	93427	14.86	1376.09	
TP2	*P. falciparum*	67	12	2/16.7	106231	5.7	40872.77	
cDPK	*H. sapiens*	80	19	3/15.8	27032	2.95	48538.90	
NAP 1	*P. knowlesi*	82	54	4/07.4	36398	2.21	180135.58	

1000001: Carboxy-terminus phosphatase domain of protein tyrosine phosphatase (2NV5), DHFR: Dihydrofolate reductase, UCE: Ubiquitin conjugating enzyme, TrpRS: Tryptophanyl tRNA synthetase, TP2: Thioredoxin peroxidase 2, 1000006: catalytic domain of protein tyrosine phosphatase (2G59), cDPK: Catalytic subunit of cAMP-dependent protein kinase, NAP1: Nucleosome assembly protein 1. ^a^Positive thermal shift is indicated by the notation + ve. *K*_D_ indicates dissociation constants. ^b^The dissociation constant reported in this table are computed from the thermal shifts obtained. *The values reported in this table are experimental *in-vitro* values.

**Table 2:** **Antimicrobial and anticancer activities of a representative set of small-molecules**^**b**^

**Protein**^**a**^	**Identity (NSC)**	**DH5α (MIC)**	**MDREC (MIC)**	**MRSA (MIC)**	**VREF (MIC)**	**HCT-116 (IC-50)**
DHFR	309401	7.813	125	31.25	31.25	0.130
	740*	ND	ND	ND	500	0.048
	339578*	62.5	250	31.25	31.25	6.11
	382035*	ND	ND	31.25	31.25	0.182
	754230*	ND	ND	ND	ND	<<0.031
1000001	111552	NA	NA	NA	NA	2.2
	246131^@^	NA	NA	NA	NA	0.024
	30205	NA	NA	NA	NA	0.146
	88882	NA	NA	N A	NA	4.44
	106863	NA	NA	NA	NA	14.5
1000006	92794	NA	NA	NA	NA	9.78
TrpRS	750690^¥^	NA	NA	NA	NA	1.11
	88882	NA	NA	NA	NA	4.44
	37168	NA	NA	NA	NA	1.34

*Reported inhibitors of DHFR independently picked up by our predictions and validated experimentally. ^@^Small molecule with known anti-cancer properties (valrubicin). ^¥^Small molecules with known anticancer properties (Sunitinib), MIC: Minimum inhibitory concentration required for 90% clearance, μg/mL units. ND: No significant inhibition. NA: not applicable. DH5α: *E. coli* strain DH5α, MRSA: Methicillin-resistant *S. aureus*, MDREC: Multi-drug resistant *E. coli*, VREF: Vancomycin-resistant *E. faecium*, HCT-116: Colon carcinoma cell line. IC-50: inhibitory concentration for 50% growth inhibition, μg/mL units. ^a^For additional details, see legend from Table 1. ^b^The values reported in this table are experimental *in-vitro* values.

### *E. coli* dihydrofolate reductase (DHFR)

*In silico* screening of *E. coli DHFR* was carried out with FINDSITE^comb^ in benchmarking mode (Additional file 1: Table S2A). The top 1% of predictions, with 83 small-molecules, was assessed for binding (Table 1). Fifteen ligands, representing 47% of interpretable curves, showed binding (Figure 1 and Table 1). Of these 15 hits, representing μM or better binders, six were previously reported inhibitors of DHFRs from various organisms [15-19]. Among these known binding molecules, methotrexate (NSC740) showed the maximum thermal shift of ~30°C followed by 7H-Pyrrolo(3,2-f) quinazoline-1, 3-diamine (NSC339578) [15], methylbezoprim (NSC382035) [16], pralatrexate (NSC754230) [17], pemetrexed (NSC698037) [18] and 6,7-bis(4-aminophenyl) pteridine-2,4-diamine (NSC61642) [19]. The approximate dissociation constant (*K*_D_) of 62 nM for the enzyme-methotrexate (NSC740) complex matches reported literature values, which range from 2 to 50 nM [20-22], within experimental error. Thus, the thermal shift methodology provides an approximate *K*_D_. The five other known inhibitors bind DHFR with low μM or nM *K*_D_s (Additional file 1: Table S3).

Nine small molecules are novel hits with no reported binding to/activity against DHFRs. These molecules are chemically diverse. The 15 different hits cluster into 10 distinct chemical classes based on a Tanimoto coefficient (TC) cutoff of 0.7 (Additional file 1: Figure S2A). NSC309401, the top novel hit in Table 1, showed apparently better binding to *E. coli* DHFR than methotrexate (K_D_ of 48 nM and a thermal shift of almost 31 degrees) and showed inhibition against several antibiotic-resistant microbial strains (Table 2). It displayed a promising MIC of 7.8 μg/mL against *E. coli* DH5α and a reasonable MIC (31.25 μg/mL) against MRSA and VREF*.* It also has very potent activity against the HCT-116 colon carcinoma cell line with an IC-50 of 0.13 μg/mL (Table 2).

This corroborates findings from the NCI human tumor cell line growth inhibition assay showing that this molecule has activity (potency not revealed) against several cancer cell lines including melanoma, prostrate, colon, and breast (http://pubchem.ncbi.nlm.nih.gov, CID: 24198955, substance SID: 573494, compound name: MLS002701801) [23]. We posit that its activity is at least partly due to DHFR inhibition. Since NSC309401 inhibits both prokaryotic and eukaryotic systems, it might be a broad specificity antifolate. 2, 4-diaminoquinazolines and their derivatives are known to inhibit DHFR (a prominent example is trimetrexate) (Rosowsky, et al., 1995) but their structures are different from NSC309401, a 7-[(4-aminophenyl) methyl]-7Hpyrrolo [3, 2-f] quinazoline-1, 3-diamine, in that the latter compound has a novel tricyclic heterocycle.

Another interesting small molecule, with no previously reported binding to DHFR, was NSC80735, with a *K*_D_ of 1.7 μM and a MIC of 10.9 μg/mL against HCT-116 (Additional file 1: Table S3). The other novel hits had affinities ranging from 6-75 μM; these hits represent potential compounds that could be improved to increase their medical significance vis-à-vis DHFR inhibition. A single novel hit had a poor affinity of ~460 μM.

### Protein tyrosine phosphatases (PTP)

The top 1% of VLS predictions (Additional file 1: Table S2B and S2C), representing 86 and 59 molecules, were tested on PTPs 1000001 and 1000006, respectively. Ten molecules, 24% of the interpretable curves, showed positive shifts for PTP 1000001, and six molecules, 14% of the interpretable curves, showed positive shifts for PTP 1000006 (see Figure 1 and Table 1). However, it should be noted here that a few of the reported molecules have low Q values representing poor signal compared to the thermal unfolding curve of the protein alone (see Materials and Methods) (Additional file 1: Table S4). All these compounds are novel hits, with no reported binding to/activity against PTPs. At a TC cutoff of 0.7, the 10 ligands showing experimental binding to 1000001 clustered into eight different subgroups (Additional file 1: Figure S2B), while the six ligands showing experimental binding to 1000006 clustered into four different subgroups (Additional file 1: Figure S2C). This again demonstrates the diversity of ligands selected by FINDSITE^comb^. Next, 32 predictions ranked below the top 1% from VLS were randomly selected and tested experimentally on 1000001 and 1000006 to demonstrate that the obtained hit rate for the top 1% was appreciably better than the background. Convincingly, as inferred by the lack of shift in *T*_m_, none showed any binding.

Among the ten hits for 1000001, seven had μM affinities, three had nM affinities with the compound NSC134137 showing a maximal thermal shift of ~12°C. This translates into an approximate *K*_D_ of 406 nM (Additional file 1: Table S4). Five of these compounds, 50% of the hits, displayed cytotoxic activity against HCT116. Valrubicin (NSC246131), (a known anticancer agent that intercalates with DNA [24]), was also shown to bind to PTP1000001 with an approximate dissociation constant of 1.5 μM. NSC246131 binding to PTP 100001 hints at promiscuity of this molecule. Three hits, NSC111552, NSC30205 and NSC88882 also showed potent cytotoxic activity (IC-50 of 2.20 μg/mL, 0.15 μg/mL and 4.44 μg/mL, respectively), while NSC106863 showed reasonable cytotoxic activity with an IC-50 of 14.5 μg/mL against the HCT-116 colon carcinoma cell line (see Table 2; Additional file 1: Table S4). We note that a single paper reports the cytotoxic activity of NSC111552 derivatives against cancer cell lines [25]. While there is no literature describing the anticancer properties of either NSC30205 or NSC88882, 9-aminoacridine-based compounds are known to be cytotoxic towards cancer cell lines [26-33]. Thus, the mode of action of NSC30205 could be similar [31]. We also posit that the PTP human homologue is one of the targets responsible for the cytotoxic activities of these molecules.

All six hits for 1000006 have apparent *K*_D_s that range from 168 nM-271.5 μM (Additional file 1: Table S4). The top hit was NSC133351 with an approximate dissociation constant of 168.3 nM. NSC92794, with a *K*_*D*_ of 161.9 μM, displayed reasonable cytotoxic activity with an IC-50 value of 9.8 μg/mL against HCT-116 colon carcinoma cell line. None of the other hits of 1000006 displayed discernible cytotoxic activity. Since 1000001 and 1000006 are both PTPs and share substantial structural similarity, there were instances where 1000001 binders also bind 1000006 (Additional file 1: Table S6 and SI).

### Ubiquitin-modifying enzyme (UCE)

For *P. falciparum* UCE, 80 molecules from the top 1% of FINDSITE^comb^ predictions (Additional file 1: Table S2G), were experimentally tested for binding (Table 1); only 51 gave interpretable thermal shift curves. Two molecules, 4% of the interpretable curves, showed binding (see Figure 1 and Table 1). NSC93427 binds to UCE, with a thermal shift of ~15°C that translates into an approximate *K*_D_ of 1.4 μM. Another compound, NSC50651, showed an apparent *K*_*D*_ of 197 μM (Additional file 1: Table S5). Future studies to assess the inhibition of *in vitro* cultures of *P. falciparum* by these small-molecules are needed to establish their utility as lead compounds for malaria treatment.

### Tryptophanyl tRNA synthetase (TrpRS)

For TrpRS, 94 compounds from the top 1% of the VLS (Additional file 1: Table S2D) were experimentally screened (Table 1). Five, constituting 42% of the interpretable curves, showed thermal shifts (see Figure 1 and Table 1). The ligands clustered into three different subgroups (Additional file 1: Figure S2D) based on a TC cutoff of 0.7. The most interesting small-molecule that binds TrpRS was Sunitinib (NSC 750690) with an approximate *K*_D_ of 1.3 μM and an IC-50 of 1.1 μg/mL for HCT-116. The observed effect might be due to its inhibition of multiple targets (receptor tyrosine kinases are known Sunitinib targets [34]).

Two other small molecules, NSC88882 and NSC37168, with ~ *K*_D_s of 3.8 μM and 9.1 μM respectively, also showed potent inhibition of HCT-116, with IC-50s of 4.44 μg/mL and 1.34 μg/mL, respectively (Table 2). NSC88882 has been shown to possess activity in the several bioassay trials undertaken by the NCI suggesting high promiscuity across several protein targets (http://pubchem.ncbi.nlm.nih.gov/, substance SID: 26665273, CID: 68249) [31]. NSC37168 also binds multiple targets within different cell types [3,35]. However, none of these reports suggest binding/inhibition of TrpRS. Other compounds that bind TrpRS were NSC50690 and NSC55152, having *K*_D_s of 7.7 μM and 39.6 μM, respectively (Additional file 1: Table S4).

### Thioredoxin peroxidase2 (TP2), cAMP-dependent protein kinase (cDPK) and nucleosome assembly protein 1(NAP1)

TP2 from *P. falciparum*, the catalytic domain of the cDPK from *H. sapiens* and NAP1 from *P. knowlesi* were tested with moderate success. Their thermal melt assay results are collated in Table 1 and Additional file 1: Table S5, with additional VLS results summarized in Additional file 1: Table S2F, S2E and S2H, respectively. Experimental thermal melt curves are shown in Figure 1. As can be seen in Additional file 1: Table S5, all these small-molecules bind with μM affinities (ranging from 41 μM-371.5 μM), making a few of them potential candidates for further development.

## Discussion

In this paper, we describe the large-scale experimental validation of the FINDSITE^comb^ VLS methodology and demonstrate that the approach is applicable to a wide variety of proteins. In contrast, previous instances of VLS coupled to experimental screening of ligands reported in the literature mostly concentrate on either a single enzyme or a single enzyme family [36-41]. FINDSITE^comb^, being a hybrid of structure-based and ligand-based VLS approaches, has many advantages: It identifies a structurally diverse set of ligands as potential hits, retains the speed of traditional ligand-based approaches, and removes the requirement of traditional structure-based approaches that a high-resolution structure of the protein target of interest be solved. Thus, ~75% of a given proteome is accessible to this VLS methodology. This affords the possibility not only of identifying novel hits, but also for repurposing FDA approved drugs, and concomitantly suggesting possible drug side effects.

Demonstration of the methodology on a diverse set of proteins with differing folds suggests that the method is a general and effective approach to discovering novel protein-ligand binding interactions. The primary success rates of 4%-47% are dramatic when compared to rates reported in the literature. Since only a tiny fraction of the protein/ligand binding predictions were assessed experimentally (20-50 of the top ranked predictions from FINDSITE^comb^), these success rates are even more significant than the raw numbers would suggest. For instance, in another study describing the HTS of a diverse library of 50,000 small-molecules against *E. coli* DHFR, the primary hit rate was 0.12% [14], whereas 47% of the 32 molecules predicted by FINDSITE^comb^ bind with μM affinities or better. Indeed, the finding that many ligands have *K*_D_s in the nM and μM range is encouraging. For three different proteins, novel nM binders were identified. Demonstration of antibacterial and cytotoxic activity by some of these compounds further suggests that the present methodology is a promising approach to identify novel hits and could help enrich the drug discovery pipeline. However, we are aware that hits generated through thermal-shift methodology relying on an extrinsic fluorophore will require additional validation.

Not only has a methodological advance been demonstrated, but also the results hold possible medical significance. We have identified several interesting hits that might represent starting scaffolds for drug design for a number of clinically important protein targets. For example, DHFR, a pivotal enzyme in the nucleotide biosynthetic pathway in *E. coli *[42] evolves resistance to available inhibitors by several mechanisms [43,44]. This is a major problem because drug-resistant *E. coli* causes the highest number of infections in hospitalized patients [35]. Thus, there is an urgent need to identify novel potent inhibitors of DHFR. In that regard, the current study provides nine novel structurally diverse small-molecule binders with apparent affinities ranging from nM to μM that are interesting hits that could be developed as lead molecules for *E. coli* DHFR inhibition. By assessing the potential of these ligands against a diverse set of drug-resistant microbial strains and colon cancer cells, we established the range of effectiveness of these compounds. A potent antibacterial and 7 molecules with cytotoxic effect against HCT-116 colon carcinoma cell line were found. This information can be exploited in designing species-specific inhibitors. Yet other examples are the pathogens *P. falciparum*, which causes malignant malaria in humans, and *P. knowlesi*, implicated in an emergent form of malaria that can infect humans [45]. Rapid evolution of resistance to known antimalarials is a major issue [46]. The present study yielded 8 hits to three different enzymes that carry out critical processes of ubiquitin-mediated post-translational modification (UCE) [47], oxidative protection of the parasite during its intraerythrocytic stages (TP2) [48] and histone transport & chromatin assembly (NAP1) [49], in the pathogen. Finally, four distinct target proteins representing members of three families, tRNA synthetases [50], phosphatases and kinases [51,52] implicated in diseases such as cancer, were examined with 24 novel protein-ligand binding interactions reported. Interestingly, these studies also identified unanticipated binding interactions of well-known drugs with alternative targets. Sunitinib, a well-documented inhibitor of receptor tyrosine kinases [34], binds to TrpRS with high-affinity. This reinforces the belief that drug molecules, at least partly, work by interfering with the function of multiple targets within the cellular milieu. It is well known that developing a new drug is a time consuming and expensive process that can take 12–15 years. Such off-target interactions could be exploited towards repurposing available drugs for alternative protein targets, thus reducing the cost and time duration of drug-discovery.

## Conclusions

In conclusion, we have demonstrated that FINDSITE^comb^ is an automated, robust and rapid methodology that can identify novel protein-ligand binding interactions that are often in the nM range or better, and which, in combination with appropriate mechanistic studies and biological activity assays can be a promising tool for lead identification/drug discovery. The presented results show that predicted structures can be successfully used for virtual ligand screening, and by exploiting the ideas of LHM, diverse novel small molecule binders can be identified even when the closest template is distantly related to the protein target of interest. Since medically relevant proteins often have a large number of evolutionarily related solved, holo protein structures that can serve as templates, they are a particularly good class of targets for the present methodology. However, we note that the methodology also works when there are few solved holo templates structures in the PDB, e.g. for GPCRs [12]. Work is now in progress to extend and experimentally validate the approach on a broader class of proteins and small molecule ligands.

## Methods

### Details about reagents are provided in SI

**Figure 2:**
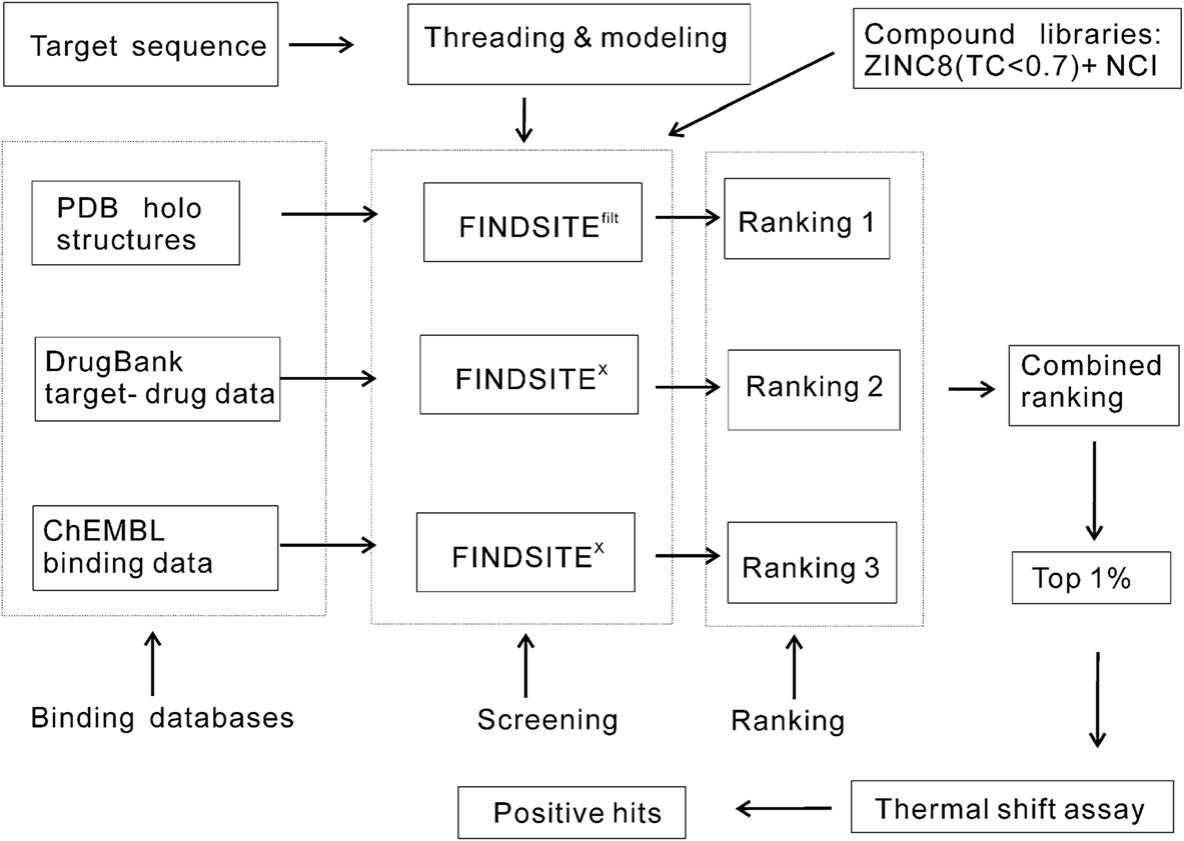
**Flowchart of FINDSITE**^**comb**^**.**

Figure 2 shows the flowchart of FINDSITE^comb^ methodology [12] in combination with experimental validation protocol. FINDSITE^comb^ is a composite approach consisting of the improved FINDSITE-based approach [9] FINDSITE^filt^ and the extended FINDSITE-based approach FINDSITE^X ^[53]. In what follows, we detail the two FINDSITE-based component approaches and their benchmarking and prediction results.

### FINDSITE^filt^ for ligand virtual screening using experimental bound structures

**Figure 3:**
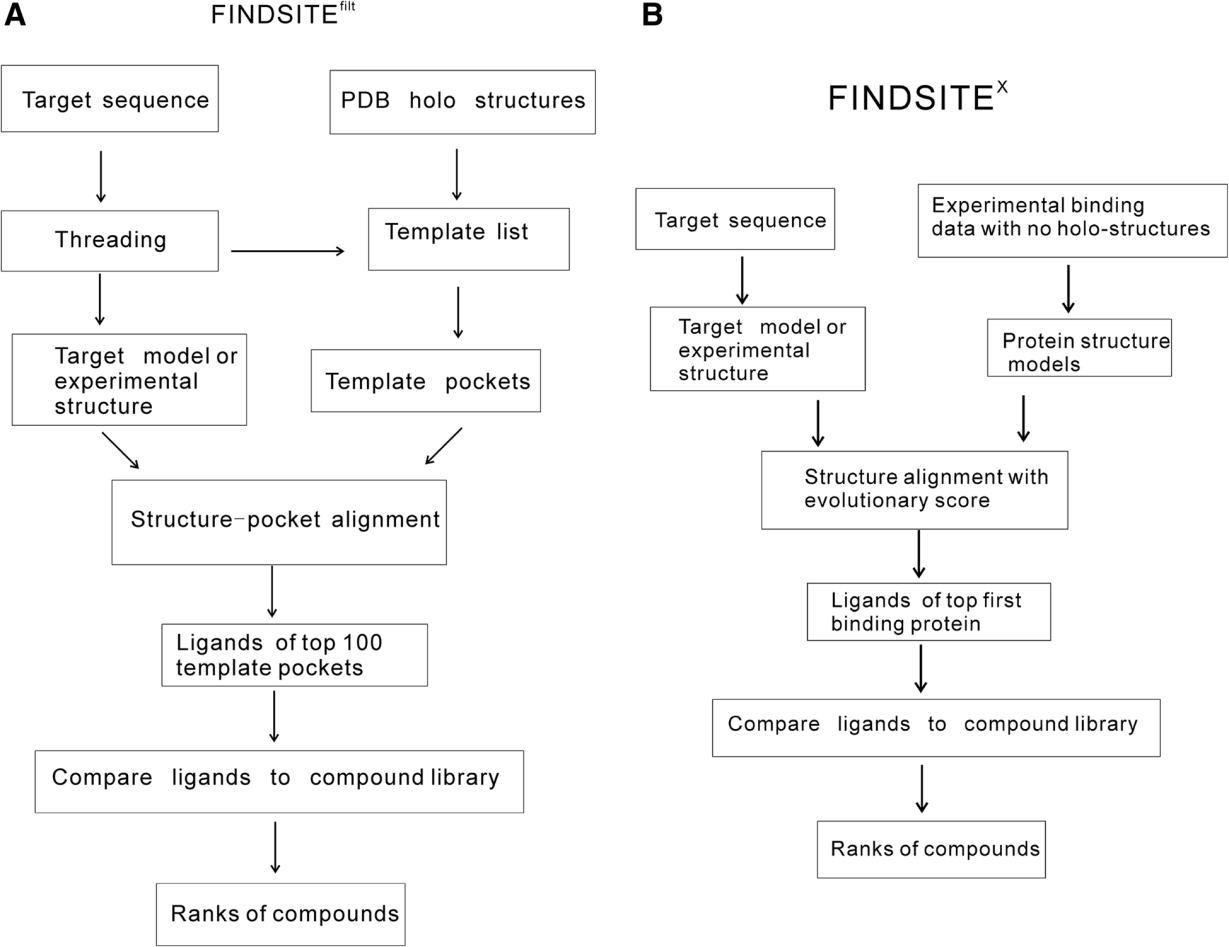
**Flowchart of two FINDSITE-based component approaches (A) FINDSITE**^**filt**^** (B) FINDSITE**^**X**^**.**

The FINDSITE^filt^ flowchart is shown in Figure 3(A) and consists mainly of three steps: (A) Finding a sub-set of protein template in the library of holo PDB structures (experimental structures with bound ligands) that are putatively evolutionarily related to the target using target sequence and threading approaches; (B) Filtering the sub-set of holo PDB structures using the target structure (experimental or modeled) and structure comparison methods; (C) Selecting pockets and ligands from the filtered sub-set for binding site and virtual screening predictions.

FINDSITE^filt ^[12] employs a heuristic structure-pocket alignment procedure and a sequence dependent scoring function to rank the holo templates in step (B) above. The alignment is evaluated using the sequence dependent score:

(1) $$\mathrm{SP}-\mathrm{score}=\sum_{\mathrm{aligned}\;\mathrm{residue}\;a,b}\mathrm{BLOSUM}62\left(a,b\right),$$

where BLOSUM62(a,b) is the BLOSUM62 substitution matrix [54]. Templates are ranked by their *SP*-scores and the ligands corresponding to the top 100 templates are selected as template ligands for ligand virtual screening.

### FINDSITE^X^ for ligand virtual screening using experimental binding data without bound structures

FINDSITE^filt^’s performance relies on the existence of a sufficient number of holo PDB structures homologous to the target. This is not true for most membrane proteins where even apo structures (structures without bound ligands) are rare. Thus, for some of the most interesting drug targets, such as the G-Protein Coupled Receptors (GPCRs) and ion-channels, FINDSITE^filt^ has limited performance. The FINDSITE^X^ approach [53] was developed to overcome the shortcomings of FINDSITE^filt^ on these kinds of targets. The flowchart of FINDSITE^X^ is shown in Figure 3(B). FINDSITE^X^ utilizes experimental binding data without ligand bound experimental structures. To use the benefits from structure comparison, structures of proteins in experimental ligand binding database are modeled. FINDSITE^X^ uses the fast version of the structure modeling approach TASSER^VMT ^[55] (TASSER^VMT^-lite [53]) to create a virtual library of protein-ligand structures analogous to the PDB holo structures but without experimentally solved protein-ligand complex structures. Since there is no reliable pocket information for the virtual holo structure, whole structure comparison of the target to the templates (in the virtual holo structures) using fr-TM-align [56] is used. To reduce false positives, especially for targets like GPCRs where almost all structures are similar (TM-score > 0.4), a sequence dependent score similar to the SP-score in Eq. (1) over the fr-TM-aligned residues is used instead of the TM-score. The ligands of the top ranked templates are used as template ligands for searching against compound library. To identify template-ligand pairs, the DrugBank drug-target relational database [57] and the ChEMBL bioactivity database [58] are used.

### FINDSITE^comb^ for ligand virtual screening

FINDSITE^comb^ is the combination of FINDSITE^filt^ that uses holo PDB structures as templates and FINDSITE^X^ that utilizes two independent ligand binding databases. For a given target and compound library, if there is no target structure input, TASSER^VMT^-lite [53] models the structure. Then, three independent virtual ligand screening runs are conducted: (a) FINDSITE^filt^ using the holo PDB structure library; (b) FINDSITE^X^ using the DrugBank virtual holo structure library; and (c) FINDSITE^X^ using the ChEMBL virtual holo structure library. For each virtual screening library, the following score is used to measure the likelihood of a compound to be a true compound of the target:

(2) $$\begin{array}{l} \mathrm{mTC}=w\frac{\sum_{l=1}^{N_{\mathrm{lg}}}\mathrm{TC}\left(L_{l},L_{\mathrm{lib}}\right)}{N_{\mathrm{lg}}}\\ \;+\left(1-w\right)\max_{l\in \left(1,\ldots ,N_{\mathrm{lg}}\right)}\left(\mathrm{TC}\left(L_{l},L_{\mathrm{lib}}\right)\right), \end{array}$$

where TC stands for the Tanimoto Coefficient [59], *N*_lg_ is the number of template ligands from the putative evolutionarily related proteins; *L*_*l*_ and *L*_*lib*_ stand for the template ligand and the ligand in the compound library, respectively; *w* is a weight parameter. The first term is the average TC [11]. The second term is the maximal TC between a given compound and all the template ligands. Here, we empirically choose *w* = 0.1 to give more weight to the second term so that when the template ligands are true ligands of the target, they will be favored. For a given compound, three independent virtual screenings give three *mTC* scores and the maximal score is used for the combined ranking.

In this study, to experimentally validate FINDSITE^comb^ under non-trivial conditions, i.e. there are no close homologous templates to the target, we have excluded all templates having sequence identity > 30% to given target in the PDB holo structures, DrugBank targets and ChEMBL targets.

### Comparison of FINDSITE^comb^ to traditional docking-based methods

We previously conducted a benchmarking test of FINDSITE^comb^ on the DUD set (A Directory of Useful Decoys set [60]) and compared our results to the state-of-the-art docking-based methods for ligand virtual screening. The DUD set is designed to help test docking algorithms by providing challenging decoys. It has a total of 2,950 active compounds and a total of 40 protein targets. For each active, there are 36 decoys with similar physical properties (e.g. molecular weight, calculated LogP) but dissimilar topology. Two freely available traditional docking methods AUTODOCK Vina [61] (http://vina.scripps.edu/) and DOCK 6 [62] (http://dock.compbio.ucsf.edu/DOCK_6/) were compared to FINDSITE^comb^. AUTODOCK Vina was tested on the DUD set and shown to be a strong competitor against some commercially distributed docking programs (http://docking.utmb.edu/dudresults/). DOCK 6 is an update of the DOCK 4 program [62]. These two methods represent state-of-the-art traditional docking-based approaches that are computationally expensive, but do not require a known set of binders for a given target as opposing to traditional ligand similarity-based approaches. FINDSITE^comb^ also does not require a known set of binders for the target, but is an order of magnitude faster than docking methods. Most importantly, FINDSITE^comb^ does not require a high-resolution experimental structure of the target. Thus, it is applicable for screening both large compound library and for genomic scale targets.

The performance of a given approach for virtual screening is evaluated by the Enrichment Factor (EF) within the top *x* fraction (or 100*x*%) of the screened library compounds defined as:

(3) $$EF_{x}=\frac{\mathrm{Number}\;\mathrm{of}\;\mathrm{true}\;\mathrm{positives}\;\mathrm{within}\;\mathrm{top}\;100x\%}{\mathrm{Total}\;\mathrm{number}\;\mathrm{of}\;\mathrm{true}\;\mathrm{positives}\times x}.$$

A true positive is defined as an experimentally known binding ligand/drug or one that has a TC = 1 to an experimentally validated binding ligand/drug. For *x* = 0.01, EF_0.01_ ranges from 0 to 100 (100 means that all true positives are within the top 1% of the compound library). Another evaluation quantity employed here is the AUAC (area under accumulative curve of the fraction of true positives versus the fraction of the screened library).

The performance of the three approaches on the DUD set using experimental target structures is shown in Table 3. FINDSITE^comb^ shows about 3 times the EF_0.01_ of AUTODOCK Vina or DOCK 6 for the top 1% selected compounds, with an EF_0.01_ of 13.4 versus 4.80 and 3.72, respectively. FINDSITE^comb^ has significantly better overall performance in terms of its AUAC (0.774 vs. 0.586 and 0.426). Although we do not have direct access to some of the commercially available approaches compared in Ref. [63], we note that FINDSITE^comb^ has a better AUAC than the best performing GLIDE (v4.5) [64,65] (mean AUAC = 0.72) and all other compared methods: DOCK 6 (mean AUAC = 0.55), FlexX [66] (mean AUAC = 0.61), ICM [67,68] (mean AUAC = 0.63), PhDOCK [69,70] (mean AUAC = 0.59) and Surflex [71-73] (mean AUAC = 0.66) [63]. The results of DOCK 6 in Ref [63] are better than that in Table 3 is due to the use of flexible docking and expertise in input preparation in Ref. [63], whereas here we employed default input and rigid docking.

**Table 3:** Performance of methods on the 40 protein DUD set using experimental structures

**Method**	**Average EF**_**0.01**_	**Average EF**_**0.05**_	**Average EF**_**0.1**_	**Average AUAC**
FINDSITE^comb^	13.4	6.56	4.37	0.774
AUTODOCK Vina	4.80	3.01	2.40	0.586
	(5.3×10^-4^)^a^	(9.4×10^-4^)	(7.7×10^-4^)	(3.0×10^-7^)
DOCK 6	3.72	1.79	1.24	0.426
	(1.5×10^-4^)	(1.8×10^-5^)	(9.9×10^-7^)	(1.3×10^-12^)

^a^Numbers in parentheses are two-sided p-values of Student-*t* test between FINDSITE^comb^ and docking methods.

We next examined the effect of target structure quality on the performance of methods. In Table 4, we show the enrichment factors EF_0.01_ and EF_0.1_ of different methods using experimental and modeled target structures for a subset of 30 targets from DUD set. The other 10 targets are not included because the modeled structures have extended long tails (not compact) and their dimensions are too large for docking methods. The results of FINDSITE^comb^ change very little when modeled structures as compared to experimental structures are used. This is not the case for either DOCK6 or AUTODOCK whose performance significantly deteriorates.

**Table 4:** Comparison of methods for the 30 protein DUD set using experimental and modeled structures

**Method**	**Ave. EF**_**0.01 **_**(expt. structure)**	**Ave. EF**_**0.01 **_**(modeled structure)**	**Ave. EF**_**0.1 **_**(expt. structure)**	**Ave. EF**_**0.1 **_**(modeled structure)**
FINDSITE^comb^	14.1	13.3	4.54	4.53
AUTODOCK Vina	5.45	2.39	2.48	1.40
DOCK 6	3.82	3.05	1.29	0.87

### Large scale testing of FINDSITE^comb^ on generic drug targets

Since FINDSITE^comb^ is much faster than traditional docking approaches and can use modeled as well as experimental structures, we can perform large-scale testing on drug targets (some of which lack experimental structures). This kind of test is not feasible for traditional docking methods. We tested FINDSITE^comb^ on a set of 3,576 DrugBank [57] targets that we can confidently model using TASSER^VMT^-lite [53]. We use modeled target structures even for those targets that have experimental PDB structures. Drugs of all the 3,576 targets are buried in a background of representative compounds that are culled to TC < 0.7 to each other from the ZINC8 library [74]. The total number of screened compounds for each target is 74,378 (6,507 drugs +67,871 ZINC8 compounds).

The test results are shown in Table 5. FINDSITE^comb^ achieves an average enrichment factor of 52 for the top 1% of (*viz.* ranked within the top 744) selected compounds; moreover, about 65% of the targets have an EF_0.01_ > 1 (EF = 1.0 is by random selection). Thus, on average about half of the true drugs of typical target will show up within top 1% of the screened compounds. FINDSITE^comb^ will be helpful in enriching true binders for 65% of the targets in a typical genome sequence. We note that FINDSITE^comb^ is better than any of its individual components. The major contribution to FINDSITE^comb^ is from FINDSITE^filt^ or holo PDB structure templates.

**Table 5:** Performance of FINDSITE methods for 3,576 drug targets

**Method (binding database)**	**Average EF**_**0.01**_	**# (%) of targets having EF**_**0.01**_ **> 1**
FINDSITE (PDB)	31.7	1526 (43%)
FINDSITE^X^ (DrugBank)	36.6	1714 (48%)
FINDSITE^X ^(ChEMBL)	9.5	566 (16%)
FINDSITE^filt^ (PDB)	46.0	2080 (58%)
FINDSITE^comb^	52.1	2333 (65%)

### Experimental validation of FINDSITE^comb^

For the experimental blind validation of this work, a compound library with molecules from the National Cancer Institute (NCI) and ZINC8 [74] (TC < 0.7) as background was used. The open chemical repository maintained by the Developmental Therapeutics Program (DTP) at NCI/NIH is a comprehensive set of small molecules consisting of compounds from the diversity set, mechanistic set, natural product set and approved oncology drug set. Compounds constituting the diversity set were derived from a parent library of ~140,000 compounds based on the following criteria: (1) Distinctness of the molecule, its pharmacophores and its conformational isomers, (2) Rigidity (5 or fewer rotatable bonds), (3) Planarity and (4) Pharmacologically desirable features. Compounds constituting the mechanistic set were selected from a seed library of 37,836 compounds tested on the NCI human tumor 60 cell line screens and represent compounds that show a broad range of growth inhibition. Compounds in the natural product set were selected from 140,000 compounds in the DTP open repository collection based on (a) origin, (b) purity, (c) structural diversity (differential scaffolds structures with varied functional groups), and (d) availability. The compounds in the approved oncology drug set consist of current FDA-approved drugs.

The reason for using NCI molecules was that they are easy to obtain. The NCI molecules are downloaded from NCI (http://dtp.nci.nih.gov/branches/dscb/repo_open.html) and consist of 1597 molecules from the Diversity Set III, 97 from the Approved Oncology Drugs Set IV, and 118 from the Natural Product Set II (total 1812 NCI molecules). The important fact is that no *a priori* target-compound binding information is used in both virtual screening and experimental validation. Together with the ZINC8 background, a total of 69683 molecules are screened by FINDSITE^comb^. NCI molecules ranked within the top 1% (i.e. higher than ~700^th^) for each target are subsequently considered for thermal shift experimental validation.

### Acquisition and quantification of thermal shift assays

High throughput thermal shift assays were carried out following established guidelines (Additional file 1: Table S1) [13,75]. Protein melting curves were obtained from samples aliquoted in 96-well plates using a RealPlex quantitative PCR instrument from Eppendorf (Eppendorf, NY, USA), with Sypro orange dye from Invitrogen as the fluorescent probe. A uniform final concentration of 5 X (supplied as a 5000 X stock solution) was used in all experiments. The dye was excited at 465 nm and emission recorded at 580 nm using the instrument’s filters. A heating ramp of 1°C/min from 25°C to 74°C was used, and one data point acquired for each degree increment. For standardization, different buffers and pH were checked. Thereafter, 100 mM HEPES pH 7.3 and 150 mM NaCl were used in all unfolding experiments. The volume of each reaction was 20 μl, and appropriate dye and protein controls were included. All experiments were done with a minimum of two replicates, with the mean value considered for further analysis. Several drugs/small molecules interact with Sypro orange and lead to aberrant signal enhancements. An additional control to rule out drug-dye interaction was carried out with all the constituents kept constant except for the protein of interest. The protein/protein-drug curves were reported after subtracting the respective dye alone/drug-dye curves.

Each melting curve was assigned a quality score (Q), the ratio of the melting-associated increase in fluorescence (ΔF_melt_) to the total fluorescence range (ΔF_total_). Q = 1 is a high-quality curve, while Q = 0 indicates no thermal transition [75]. Though an arbitrary Q value cutoff was not applied to judge curve quality, the curves were manually curated with Q values reported. A substantial fraction of ligands tested against the various proteins displayed no thermal transitions, Q = 0, or showed multi-step unfolding behavior. These were ignored (see Table 1).

### Data analysis

Subsequent to standardization, (see SI Methods), the validity of the top 1% of FINDSITE^comb^’s predictions on the test set of eight diverse proteins was examined. To be conservative, we focused only on those protein/ligand pairs showing single sigmoidal thermal transition curves. The fit to Boltzmann’s equation (Eq. 1) was employed to estimate the melting temperature from the observed intensity, *I*.

(4) $$I=I_{\min}+\frac{\left[I_{\max}-I_{\min}\right]}{1+e^{\left(\frac{\mathrm{Tm}-T}{a}\right)}}$$

*I*_*min*_ and *I*_*ma*x_ are the minimum and maximum intensities; *a* denotes the slope of the curve at the transition midpoint temperate, *T*_m _[13]. To estimate thermodynamic parameters, both van’t Hoff [76] and Gibbs-Helmholtz analyses were done [77]. To estimate the approximate ligand-binding affinity at *T*_m_, Eq. (2) from reference [78] was used with slight modifications; ΔCp is ignored.

(5) $$K_{L}\left(T_{m}\right)=\frac{\exp\left\{\frac{-\mathrm{\Delta H}}{R}\left(\frac{1}{\mathrm{Tm}}-\frac{1}{\mathrm{To}}\right)\right\}}{L}$$

*K*_L_(*T*_m_) is the ligand association constant and [L] is the free ligand concentration at *T*_m_ ([L_Tm_] ~ [L]total, when [L]_total_ > > the total concentration of protein. *K*_D_ is the inverse of *K*_L_(*T*_m_).

To eliminate the possibility of thermal shifts arising because organic molecules form colloidal aggregates [79], the complete NCI set was compared to the database of known aggregators maintained at http://advisor.bkslab.org/search/. Since the thermal shift assay is incompatible with the presence of detergents, (the method of choice to eliminate aggregation-based thermal shifts), we limited ourselves to estimate chemical similarity to known aggregators. At a stringent TC cutoff of 0.9, none of the molecules reported as possessing either binding or antimicrobial/cytotoxic activities are similar to known aggregators.

### Antimicrobial and cytotoxic assays on cancer cell lines

Antimicrobial and anti-cancer tests were performed as in [80]. DHFR binders were tested on *E. coli* DH5α [positive control: Nitrofurantion (10 mg/ml in DMSO, negative control: DMSO], multi-drug resistant *E. coli* SMS-3-4 (ATCC BAA-1743) (MDREC) [positive control: Nitrofurantion (10 mg/ml in DMSO), negative control: DMSO], methicillin-resistant *S. aureus* (ATCC 33591) (MRSA) [positive control: Vancomycin (10 mg/ml in DMSO), negative control: DMSO], vancomycin-resistant *E. faecium* (ATCC700221) (VREF) [positive control: Chloramphenicol (10 mg/ml in DMSO), negative control: DMSO], and colon carcinoma cells HCT-116 [positive control: etoposide (20 μg/ml in DMSO), negative control: DMSO]. Phosphatase (1000001 and 1000006) binders and tryptophanyl tRNA synthetase binders were tested on the colon carcinoma cell line HCT-116.

## Abbreviations

VLS: Virtual ligand screening; HTS: High throughput screening; DMSO: Dimethyl sulfoxide; PTP: Protein tyrosine phosphatase; DHFR: Dihydrofolate reductase; UCE: Ubiquitin-conjugating enzyme; TrpRS: Tryptophanyl tRNA synthetase; NAP: Nucleosome assembly protein; TP: Thioredoxin peroxidase; cDPK: Catalytic domain of cAMP-dependent protein kinase.

## Competing interests

The authors declare no competing financial interest. We are currently applying for patents relating to the content of the manuscript.

## Authors’ contributions

BS analyzed and compiled the virtual ligand screening (VLS) data, designed and carried out the experimental high-throughput thermal shift (HTS) assays, analyzed and interpreted the data, and drafted the manuscript. HZ carried out the computational VLS experiments, analyzed the VLS data, drafted the sections on VLS and critically reviewed the manuscript. JK was instrumental in designing and analyzing the antibacterial and anticancer activity assays and helped in critically reviewing the manuscript. JS conceived of the study, participated in its design and coordination, provided appropriate resources, helped analyze the data, and was involved in drafting and critically reviewing the manuscript. All authors read and approved the final manuscript.

## Supplementary Material

Additional file 1 **Detailed FINDSITE**^**comb **^**VLS results, Thermal shift assay standardization: methods and results, HTS protocol table, detailed results on the thermal shift assay and biological activity assay for the eight protein in tabular form, discussion on the differences between 1000001 and 1000006 VLS and experimental overlap and figure depicting the diversity of compounds picked up by the current methodology.** Click here for file
