# Supplementary material for: Experimental validation of FINDSITEcomb virtual ligand screening results for eight proteins yields novel nanomolar and micromolar binders
Source: J Cheminform. 2014 Apr 26;6:16. doi: 10.1186/1758-2946-6-16 (PMC4038399; doi:10.1186/1758-2946-6-16)

**SUPPLEMENTARY INFORMATION**

**MATERIALS AND METHODS**

**Reagents -** All reagents and chemicals, unless mentioned otherwise, were procured from Sigma-Aldrich (St. Louis, MO) with the following exceptions: HEPES, pH 7.3 buffer was obtained from Fischer Bioreagents and dimethyl sulfoxide (DMSO) from MP Biomedicals LLC. Sypro orange dye was obtained from Invitrogen (Carlsbad, CA). 96-well PCR-plates and plate seals were from Eppendorf (Eppendorf, NY, USA). Pig heart citrate synthase, bovine serum albumin, carbonic anhydrase I from human RBC and pig heart malic dehydrogenase were obtained from Sigma-Aldrich (St. Louis, MO). *E. coli* DHFR was a gift from Eugene Shakhnovich. The C-terminal phosphatase domain from *R. norvegicus* (PDB ID: 2NV5) (1000001) and the catalytic domain of PTP from *H. sapiens* (PDB ID: 2G59) (1000006) were gifts from the New York structural genomics consortium (NYSGC). Nucleosome assembly protein 1 from *P. knowlesi*, and thioredoxin peroxidase and ubiquitin conjugating enzyme from *P. falciparum* were gifts from the Structural Genomics for Pathogenic Protozoa (SGPP) consortium. Human tryptophanyl tRNA synthetase was a gift from Paul Schimmel. Human cDPK was a gift from Susan Taylor. Small molecules constituting oncology drug set III (97 compounds), diversity set III (synthetic) (1597 compounds) and natural product set (120 compounds) were provided by the NCI/DTP open chemical repository (http://dtp.cancer.gov).

## Thermal-shift assays

Thermal melt assays were standardized on several protein/ligand pairs with reported *T*_m_ shifts. Thermal melt curves for citrate synthase were generated with both cognate ligand oxaloacetate (cat. no. O4126) and non-cognate (and nonbinding) NADH. The protein concentration was 5 µM. Titration of oxaloacetate (10 µM-200 µM) was carried out to establish the concentration dependence of the melting temperature. Thermal melting of malic dehydrogenase was performed with its cognate ligand NADH (cat no. N8129) and non-cognate ligand oxaloacetate. Studies of the concentration dependence of NADH (1 mM-5 mM) on melting temperature shifts for malic dehydrogenase at 5 µM and 10 µM concentrations, respectively, were also performed. Effects of non-cognate ligands NADH and oxaloacetate were ascertained with bovine serum albumin (15 µM concentration) as a control (data not shown).

Thermal unfolding of the eight benchmark proteins was carried out in a 20 μl reaction mix with 100 mM HEPES pH 7.3, 150 mM NaCl, 5 X Sypro orange dye, 10 μM protein and 1 mM of all the predicted ligands.

**RESULTS**

**A) *In silico* predictions:**

***E. coli* dihydrofolate reductase**: The structure was modeled on an ensemble of 20 different templates; the best was the PDB entry 3DFR (27% SID). The top first templates used to identify template ligands by FINDSITE^filt^ and FINDSITE^X^ are: *3CSEA* from PDB with 27% sequence identity to the target; *Dihydrofolate reductase* with sequence identity of 30% to the target from DrugBank; a cell division protein *FtsZ (Mycobacterium tuberculosis)* with a sequence identity of 15% to the target from ChEMBL. The 106 NCI molecules within the top 1% (700) are listed in Table S2A along with their source (template whose ligands are closest to the molecules measured by Eq.(2)). The predicted 32 binding residues are: 5ILE 6ALA 7ALA 19ALA 20MET 27ASP 28LEU 31PHE 32LYS 43GLY 44ARG 45HIS 46THR 50ILE 54LEU 57ARG 62LEU 63SER 64SER 65GLN 76LYS 77SER 94ILE 95GLY 96GLY 97GLY 98ARG 99VAL 100TYR 102GLN 113THR 123THR.

***R. norvegicus* protein tyrosine phosphatase:** The sequence of 1000001, the carboxy-terminal domain of PTP delta family 2A from *R. norvegicus*, was modeled on an ensemble of 20 templates; the best was 2OC3 (SID 30%). The top templates used to identify template ligands by FINDSITE^filt^ and FINDSITE^X^ are: *3BLUA* from PDB (sequence identity 21%); *Tyrosine-protein phosphatase yopH* from DrugBank (19%); *Protein-tyrosine phosphatase 1 (Saccharomyces cerevisiae S288c)* from ChEMBL (29%). The 90 predicted NCI molecules within the top 1% are listed in Table S2B. The predicted binding residues: 56TYR 58ASN 59VAL 223CYS 224SER 225ALA 226GLY 227VAL 228GLY 229ARG 264TYR 267GLN 271GLN.

***H. sapiens* protein tyrosine phosphatase:** The sequence of 1000006, a receptor PTP type O family 3 from *H. sapiens*, was modeled on an ensemble of 20 templates; the best was 2I1Y (SID 28%). The top templates used to identify template ligands by FINDSITE^filt^ and FINDSITE^X^ are: *3BLTA* from PDB (24%); *Tyrosine-protein phosphatase yopH* from DrugBank (15%); *Secreted protein-tyrosine phosphatase (Yersinia pestis)* from ChEMBL (15%). 60 NCI molecules within the top 1% of the screened compound library are given in Table S2C. Predicted binding residues are 55TYR 57ASN 58ILE 133LYS 187TRP 223CYS 224SER 225ALA 226GLY 227VAL 228GLY 229ARG 263MET 264SER 267GLN 271GLN. This binding site is slightly larger than that of Phosphatase_rat. (16 versus 13 residues). 1000001 and 1000006 share 41% SID. Not surprisingly, the overall features of both modeled protein structures were similar, including the pocket of interest.

***H. sapiens* tryptophanyl tRNA synthetase:** The sequence of W-tRNA synthetase from *H. sapiens* was modeled on an ensemble of 20 structures; the best was 3hzr (SID 27%). Table S2D lists the 107 NCI molecules within the top ranked 1%. The predicted binding residues are: 71SER 72ASN 73HIS 159TYR 160THR 161GLY 162ARG 163GLY 169MET 170HIS 172GLY 173HIS 176PRO 194GLN 196THR 199GLU 280PHE 284GLN 307ILE 309CYS 310ALA 312ASP 313GLN 317PHE 338THR 339PHE 340PHE 350MET.

***H. sapiens* catalytic domain of cAMP-dependent kinase:** The top templates used to identify template ligands by FINDSITE^filt^ and FINDSITE^X^ are: *3DFCB* from PDB (29%); *Serine/threonine-protein kinase 6* from DrugBank (27%); *Serine/threonine-protein kinase Aurora-A (Mus musculus)* from ChEMBL (24%). NCI molecules are listed in Table S2E. The predicted binding residues are: 50LEU 51GLY 52THR 53GLY 54SER 55PHE 56GLY 57ARG 58VAL 71ALA 73LYS 75LEU 105VAL 121MET 122GLU 123TYR 124VAL 128GLU 169LYS 171GLU 174LEU 184THR 185ASP 188PHE 328PHE.

***P. falciparum* thioredoxin peroxidase:** The top templates used to identify template ligands by FINDSITE^filt^ and FINDSITE^X^ are: *3A2WC* from PDB (24%); *Bacterioferritin co-migratory protein* from DrugBank (20%); *Nicastrin(human)* from ChEMBL (14%). Table S2F lists the predicted 68 NCI molecules within the top ranked 1%. The predicted binding residues are: 60PRO 64THR 65PHE 66VAL 67CYS 142ARG 161LEU.

***P. falciparum* Ubiquitin conjugating enzyme:** The top templates used to identify template ligands by FINDSITE^filt^ and FINDSITE^X^ are: *3O2UB* from PDB (27%); *Glutathione peroxidase* from DrugBank (15%); *Myotonin-protein kinase* from ChEMBL (12%). Table S2G lists the predicted 80 NCI molecules within the top ranked 1%. The predicted binding residues are: 65PRO 66LYS 67ILE 68ILE 83ALA 84ILE 89LEU.

***P. knowlesi* nucleosome assembly protein 1:** The top templates used to identify template ligands by FINDSITE^filt^ and FINDSITE^X^ are: *2E50A* from PDB (22%); Outer *membrane protein C* from DrugBank (13%); *Nucleophosmin (human)* from ChEMBL (10%). Table S2H lists the predicted 86 NCI molecules within the top ranked 1%. The predicted binding residues are: 3THR 4THR 5GLU 157PHE; they are metal binding sites.

**B) Standardization:**

Standardizations were carried out on the carbonic anhydrase/TFMSA, citrate synthase/oxaloacetate and malate dehydrogenase/ NADH pairs (Fig S1A). Ligand titrations were also carried out on the standard proteins to demonstrate the concentration dependence of ligands on *T*_m_ shifts (Fig S1 B-D). The melting temperature shift reported for carbonic anhydrase at 2000 μM TFMSA is 13.2 °C using ANS as an extrinsic fluorophore reporter dye [[11](#_ENREF_11)]. Yet another reference provides a *T*_m_ shift of approximately 8 °C at 500 μM TFMSA [[12](#_ENREF_12)]. In our studies, we observe a 7.2 °C shift at 500 μM TFMSA, a 9 °C shift at 1 mM TFMSA and a 12.3 °C shift at 10 mM TFMSA using Sypro Orange as the extrinsic fluorophore dye which is within the range of reported *T*_m_ shifts for the protein-ligand pair. Moreover, the calculated approximate dissociation constants at various TFMSA concentration from our experimental thermal melt curves range from 0.77-2.29 μM, in good agreement with the reported values of the dissociation constant that range from 1.00-2.17 μM [[12](#_ENREF_12)]. The observed differences could be ascribed to a number of varying experimental parameters such as the buffer composition, nature and concentration of the extrinsic fluorophore, salt concentration, etc.

On the other hand, citrate synthase showed a maximal thermal shift of 5.9 °C at 5 mM oxaloacetate and a minimal *T*_m_ shift of 1.9 °C at 1 mM oxaloacetate. Literature sources report a far more pronounced thermal shift of 5.5 °C at 1 mM oxaloacetate[[13](#_ENREF_13)], albeit under differing assay conditions employing aggregation and differential scanning fluorimetry. The experimental parameters that mimic our assay condition reasonably closely (HEPES pH 7.5) report a thermal shift of approximately 4 °C at 100 μM oxaloacetate [[14](#_ENREF_14)]. In contrast, we were not able to record any thermal shifts at 100 μM oxaloacetate. Even our calculated dissociation constant for citrate synthase oxaloacetate pair is 574 ± 134 μM, which is off by a factor of 100 from the values reported in literature [[15](#_ENREF_15)]. A possible explanation for this discrepancy in *T*_m_ shifts and the dissociation constant could be because the enzyme was supplied with 500 μM citrate (with a *K*_m_ of 22 μM[[16](#_ENREF_16)]) and 6 mM phosphate. The reported constants of oxaloacetate for the enzyme are 3.9 ± 0.7 μM (*K*_m_ under steady state conditions), 4.5 ± 1.6 μM (*K*_d_) and 4.3 ± 1.8 μM (*K*_i_ vis-à-vis 8-hydroxypyrene) [[15](#_ENREF_15)], approximately 4 times less than those for citrate. Hence, it is highly likely that our values are an underestimation of reported values due to the competition between citrate and oxaloacetate binding.

For malate dehydrogenase, the curves were noisy and the baseline was uneven. Hence, the curves were not interpreted critically apart from the fact that the cognate ligand, NADH, brought about a shift of 11 °C at 1 mM and 17 °C at 10 mM. These values are in good agreement with those reported in literature[[13](#_ENREF_13)].

Though the Δ*T*_m_ values for all the protein-ligand pairs matched well, within experimental error, to the values reported in literature, depending on the quality and age of the protein the absolute melting temperatures were usually lower than those reported (Fig S1).

**Protein tyrosine phosphatase:**

Since the overall structures of both 1000001 and 1000006, along with the pocket of interest, were similar, it was surprising that VLS gave different small molecule hits for the two protein molecules. To understand this discrepancy, the 12 hits obtained for the protein 1000001 were tested on 1000006. Initially, to our initial surprise, seven out of 12 molecules showed unambiguous binding to 1000006, while two showed ambiguous curves with two transitions. Furthermore, three molecules failed to bring about any shifts in the thermal melting curves indicative of no binding. Table S6 summarizes the results. Notice that the molecules, in spite of binding to 1000006, are ranked very low by the algorithm. This can be attributed to the bias introduced by template selection in the benchmarking mode. In the benchmark mode of current version of FINDSITE^comb^, ligands of templates from the PDB, ChEMBL and DrugBank having > 30% SID to the target are excluded from being used as template ligands in Eq. (2). For 1000001, FINDSITE^comb^ selects "protein tyrosine phosphatase 1 (yeast)" for template ligands from the ChEMBL library that has a 29% SID to 1000001. However this protein has a 30.2% SID to 1000006. Thus, in the benchmarking mode of 1000006, FINDSITE^comb^ did not use "protein tyrosine phosphatase 1 (yeast)"; instead, it used the template "secreted protein tyrosine phosphatase (Yersinia pestis)" that has a 15% SID to 1000006. If we had used the same template, "protein tyrosine phosphatase 1 (yeast)", for both proteins, then more similar binding results are found, with the pM binder 111552 predicted to bind both tyrosine phosphatase. Based on these results, we are currently fixing this instability in the algorithm by including more than the top best ranked templates from ChEMBL and DrugBank.

**References:**

1. Brylinski M, Skolnick J: **A threading-based method (FINDSITE) for ligand-binding site prediction and functional annotation**. *Proceedings of the National Academy of Sciences of the United States of America* 2008, **105**(1):129-134.

2. Zhou H, Skolnick J: **FINDSITE(X): a structure-based, small molecule virtual screening approach with application to all identified human GPCRs**. *Molecular pharmaceutics* 2012, **9**(6):1775-1784.

3. Zhou H, Skolnick J: **FINDSITE(comb): a threading/structure-based, proteomic-scale virtual ligand screening approach**. *Journal of chemical information and modeling* 2013, **53**(1):230-240.

4. Henikoff S, Henikoff JG: **Amino acid substitution matrices from protein blocks**. *Proceedings of the National Academy of Sciences of the United States of America* 1992, **89**(22):10915-10919.

5. Zhou H, Skolnick J: **Template-based protein structure modeling using TASSER(VMT)**. *Proteins* 2011.

6. Pandit SB, Skolnick J: **Fr-TM-align: a new protein structural alignment method based on fragment alignments and the TM-score**. *BMC bioinformatics* 2008, **9**:531.

7. Okuno Y, Tamon A, Yabuuchi H, Niijima S, Minowa Y, Tonomura K, Kunimoto R, Feng C: **GLIDA: GPCR--ligand database for chemical genomics drug discovery--database and tools update**. *Nucleic acids research* 2008, **36**(Database issue):D907-912.

8. Wishart DS, Knox C, Guo AC, Shrivastava S, Hassanali M, Stothard P, Chang Z, Woolsey J: **DrugBank: a comprehensive resource for in silico drug discovery and exploration**. *Nucleic acids research* 2006, **34**(Database issue):D668-672.

9. Gaulton A, Bellis LJ, Bento AP, Chambers J, Davies M, Hersey A, Light Y, McGlinchey S, Michalovich D, Al-Lazikani B *et al*: **ChEMBL: a large-scale bioactivity database for drug discovery**. *Nucleic acids research* 2012, **40**(Database issue):D1100-1107.

10. Brylinski M, Skolnick J: **FINDSITE: a threading-based approach to ligand homology modeling**. *PLoS computational biology* 2009, **5**(6):e1000405.

11. Matulis D, Kranz JK, Salemme FR, Todd MJ: **Thermodynamic stability of carbonic anhydrase: measurements of binding affinity and stoichiometry using ThermoFluor**. *Biochemistry* 2005, **44**(13):5258-5266.

12. Baranauskiene L, Matulis D: **Intrinsic thermodynamics of ethoxzolamide inhibitor binding to human carbonic anhydrase XIII**. *BMC biophysics* 2012, **5**:12.

13. Senisterra GA, Markin E, Yamazaki K, Hui R, Vedadi M, Awrey DE: **Screening for ligands using a generic and high-throughput light-scattering-based assay**. *Journal of biomolecular screening* 2006, **11**(8):940-948.

14. Niesen FH, Berglund H, Vedadi M: **The use of differential scanning fluorimetry to detect ligand interactions that promote protein stability**. *Nature protocols* 2007, **2**(9):2212-2221.

15. Johnson JK, Srivastava DK: **Interaction of ligands with pig heart citrate synthase: conformational changes and catalysis**. *Archives of biochemistry and biophysics* 1991, **287**(2):250-256.

16. Sullivan AC, Singh M, Srere PA, Glusker JP: **Reactivity and inhibitor potential of hydroxycitrate isomers with citrate synthase, citrate lyase, and ATP citrate lyase**. *The Journal of biological chemistry* 1977, **252**(21):7583-7590.

**Table S1**.High-throughput screening summary

| **Category** | **Parameters** | **Description** |
| --- | --- | --- |
| **Assay** | Type of assay | Thermal melt |
|  | Target | 8 different proteins as listed in Table 1 |
|  | Primary measurements | Increase in fluorescence of an extrinsic fluorophore upon protein unfolding |
|  | Key reagents | Sypro orange dye |
|  | Assay protocol | See Materials and Methods |
|  | Additional comments | See Materials and Methods |
| **Library** | Library Size | VLS: 69683 compounds, experiments HTS:1814 (DTP, NCI/NIH) |
|  | Library composition | Oncology drug set IV, diversity set III and natural product set II |
|  | Source | DTP, NCI/NIH |
|  | Additional comments | See Materials and Methods. |
| **Screen** | Format | 96 well format |
|  | Concentrations(s) tested | 1 mM of each compound |
|  | Plate controls | Appropriate dye control and protein + DMSO controls |
|  | Reagent dispensing system | Manual |
|  | Detection instrument and s/w | RealPlex quantitative PCR instrument from Eppendorf |
|  | Assay validation/QC | See Materials and Methods. Q values for all the curves were computed and curves with multiple transition were eliminated. |
|  | Normalization | All the curves were normalized on a scale of 0-1. |
|  | Additional comments | See Materials and Methods. |
| **Post-HTS analysis** | Hit criteria | Shift in the midpoint of transition (*T*_m_) in the thermal melt curve along the x-axis (representing temperature) |
|  | Hit rate | See results (from 4 %- 50 %) |
|  | Additional comments | See results |

**Table S2A**. The NCI molecules in the top 1% of the screened compound library for dihydrofolate reductase *Escherichia coli*

| Rank | CAS or NSC # | Score/Source | Rank | CAS or NSC # | Score/Source |
| --- | --- | --- | --- | --- | --- |
| 2 | 59-05-2 | 0.934/DrugBank | 266 | 318799 | 0.673/PDB |
| 3 | 137281-23-3 | 0.930/DrugBank | 282 | 403374 | 0.671/PDB |
| 4 | 330753 | 0.919/ChEMBL | 297 | 3543-75-7 | 0.670/ChEMBL |
| 8 | 33069-62-4 | 0.835/ChEMBL | 306 | 57794 | 0.669/DrugBank |
| 10 | 114977-28-5 | 0.815/ChEMBL | 317 | 339578 | 0.669/DrugBank |
| 11 | 183133-96-2 | 0.810/ChEMBL | 320 | 50654 | 0.669/DrugBank |
| 18 | 125197 | 0.772/PDB | 322 | 105584 | 0.669/ChEMBL |
| 20 | 50-91-9 | 0.768/PDB | 328 | 151721 | 0.667/ChEMBL |
| 22 | 42231 | 0.762/DrugBank | 337 | 191732-72-6 | 0.667/PDB |
| 27 | 146464-95-1 | 0.752/DrugBank | 341 | 401077 | 0.666/DrugBank |
| 33 | 121182 | 0.742/PDB | 351 | 157522 | 0.666/DrugBank |
| 34 | 75607-67-9 | 0.740/DrugBank | 355 | 319012 | 0.665/DrugBank |
| 46 | 382035 | 0.731/PDB | 367 | 23248 | 0.664/ChEMBL |
| 50 | 274905 | 0.728/PDB | 375 | 27305 | 0.663/PDB |
| 51 | 106464 | 0.727/PDB | 382 | 403447 | 0.663/ChEMBL |
| 56 | 25740 | 0.721/PDB | 383 | 337783 | 0.663/ChEMBL |
| 57 | 81462 | 0.721/DrugBank | 385 | 106399 | 0.662/ChEMBL |
| 61 | 14975 | 0.718/ChEMBL | 398 | 146770 | 0.661/ChEMBL |
| 63 | 4291-63-8 | 0.718/PDB | 413 | 183319-69-9 | 0.660/DrugBank |
| 67 | 201634 | 0.714/PDB | 418 | 7524 | 0.659/ChEMBL |
| 72 | 91529 | 0.711/ChEMBL | 419 | 7524 | 0.659/ChEMBL |
| 76 | 287088 | 0.710/ChEMBL | 424 | 302289 | 0.659/ChEMBL |
| 78 | 61610 | 0.710/ChEMBL | 427 | 299514 | 0.659/ChEMBL |
| 79 | 69-74-9 | 0.709/PDB | 429 | 46385 | 0.659/PDB |
| 84 | 339555 | 0.707/ChEMBL | 466 | 515893 | 0.656/DrugBank |
| 87 | 241998 | 0.707/PDB | 491 | 85239 | 0.655/ChEMBL |
| 107 | 14974 | 0.701/ChEMBL | 506 | 76350 | 0.654/PDB |
| 114 | 270914 | 0.698/ChEMBL | 514 | 374814 | 0.654/PDB |
| 116 | 122111-03-9 | 0.697/PDB | 515 | 43409 | 0.654/PDB |
| 123 | 176503 | 0.696/ChEMBL | 519 | 87010 | 0.654/PDB |
| 125 | 118628 | 0.695/ChEMBL | 525 | 59776 | 0.654/PDB |
| 150 | 106570 | 0.691/ChEMBL | 537 | 2353-33-5 | 0.653/PDB |
| 157 | 338250 | 0.690/ChEMBL | 547 | 50690 | 0.653/DrugBank |
| 164 | 123318-82-1 | 0.688/PDB | 551 | 20586 | 0.652/DrugBank |
| 174 | 22070 | 0.686/ChEMBL | 552 | 45153 | 0.652/PDB |
| 175 | 22070 | 0.686/ChEMBL | 577 | 210236 | 0.651/ChEMBL |
| 177 | 37641 | 0.686/ChEMBL | 597 | 305222 | 0.650/ChEMBL |
| 181 | 130801 | 0.686/DrugBank | 606 | 9168 | 0.650/ChEMBL |
| 182 | 105827 | 0.686/DrugBank | 614 | 81750 | 0.650/PDB |
| 194 | 51001 | 0.684/ChEMBL | 617 | 50680 | 0.649/DrugBank |
| 197 | 61642 | 0.683/DrugBank | 618 | 122224 | 0.649/PDB |
| 204 | 280594 | 0.682/DrugBank | 628 | 379536 | 0.649/PDB |
| 209 | 319034 | 0.681/DrugBank | 641 | 150817 | 0.648/ChEMBL |
| 213 | 96491 | 0.680/DrugBank | 644 | 123458 | 0.648/DrugBank |
| 215 | 401005 | 0.680/ChEMBL | 658 | 80735 | 0.648/PDB |
| 225 | 157035 | 0.679/ChEMBL | 661 | 55152 | 0.648/ChEMBL |
| 229 | 121032-29-9 | 0.678/PDB | 662 | 80731 | 0.648/PDB |
| 236 | 63701 | 0.677/DrugBank | 680 | 14304 | 0.647/DrugBank |
| 239 | 78623 | 0.677/PDB | 682 | 141538 | 0.647/ChEMBL |
| 247 | 22847 | 0.676/PDB | 684 | 159686 | 0.646/DrugBank |
| 248 | 133071 | 0.676/PDB | 693 | 92709 | 0.646/PDB |
| 253 | 309401 | 0.675/DrugBank | 694 | 28011 | 0.646/DrugBank |
| 264 | 49701 | 0.673/DrugBank | 695 | 1014 | 0.646/DrugBank |

**Table S2B.** NCI molecules within the top 1% of the screened compound library for protein 1000001 Protein Tyrosine Phosphatase (PTP) *Rattus norvegicus*

| Rank | CAS or NSC # | Score/Source | Rank | CAS or NSC # | Score/Source |
| --- | --- | --- | --- | --- | --- |
| 8 | 51787 | 0.826/PDB | 353 | 6731 | 0.685/ChEMBL |
| 12 | 5451-09-2 | 0.811/PDB | 355 | 280594 | 0.685/PDB |
| 14 | 5426 | 0.810/ChEMBL | 358 | 15133 | 0.685/PDB |
| 15 | 284200 | 0.809/PDB | 368 | 16646 | 0.685/ChEMBL |
| 17 | 9037 | 0.808/ChEMBL | 376 | 98857 | 0.684/PDB |
| 28 | 267461 | 0.779/ChEMBL | 385 | 180964 | 0.682/ChEMBL |
| 33 | 102314 | 0.772/PDB | 386 | 41148 | 0.682/ChEMBL |
| 38 | 73482 | 0.762/PDB | 394 | 14540 | 0.682/PDB |
| 40 | 88882 | 0.760/PDB | 399 | 61888 | 0.681/PDB |
| 41 | 47619 | 0.760/PDB | 410 | 163802 | 0.680/ChEMBL |
| 52 | 1751 | 0.752/PDB | 412 | 153172 | 0.680/ChEMBL |
| 54 | 75607-67-9 | 0.749/PDB | 418 | 62840 | 0.679/PDB |
| 56 | 400770 | 0.749/ChEMBL | 425 | 9064 | 0.678/PDB |
| 76 | 101758 | 0.740/PDB | 435 | 26744 | 0.677/ChEMBL |
| 88 | 122131 | 0.734/PDB | 439 | 163920 | 0.677/PDB |
| 89 | 326757 | 0.734/ChEMBL | 448 | 177406 | 0.677/ChEMBL |
| 102 | 156563 | 0.729/ChEMBL | 454 | 121032-29-9 | 0.676/PDB |
| 110 | 7668 | 0.726/PDB | 458 | 7962 | 0.676/ChEMBL |
| 112 | 111552 | 0.725/ChEMBL | 460 | 118628 | 0.676/ChEMBL |
| 145 | 35679 | 0.720/PDB | 465 | 37627 | 0.675/ChEMBL |
| 150 | 25368 | 0.719/PDB | 474 | 129536 | 0.675/PDB |
| 155 | 102509 | 0.718/PDB | 475 | 129536 | 0.675/PDB |
| 156 | 614552 | 0.718/PDB | 477 | 9665 | 0.675/PDB |
| 166 | 25316-40-9 | 0.714/ChEMBL | 485 | 22847 | 0.674/PDB |
| 167 | 23541-50-6 | 0.714/ChEMBL | 487 | 65537 | 0.674/ChEMBL |
| 178 | 82151 | 0.712/ChEMBL | 500 | 76988 | 0.672/ChEMBL |
| 193 | 1847 | 0.709/ChEMBL | 512 | 13156 | 0.671/ChEMBL |
| 197 | 209870 | 0.708/PDB | 513 | 227309 | 0.671/PDB |
| 201 | 134137 | 0.707/ChEMBL | 519 | 21683 | 0.671/ChEMBL |
| 210 | 143491 | 0.707/ChEMBL | 528 | 56124-62-0 | 0.670/ChEMBL |
| 214 | 21603 | 0.706/PDB | 531 | 15780 | 0.670/PDB |
| 227 | 106863 | 0.704/ChEMBL | 533 | 45923 | 0.670/PDB |
| 268 | 3001 | 0.695/PDB | 534 | 298-81-7 | 0.670/PDB |
| 274 | 1614 | 0.694/PDB | 536 | 62511 | 0.669/PDB |
| 275 | 41805 | 0.694/ChEMBL | 537 | 16437 | 0.669/ChEMBL |
| 279 | 407286 | 0.693/PDB | 547 | 35611 | 0.669/PDB |
| 282 | 110899 | 0.693/PDB | 572 | 109719 | 0.667/PDB |
| 286 | 179818 | 0.692/PDB | 619 | 11150 | 0.664/ChEMBL |
| 298 | 2561 | 0.691/PDB | 639 | 25740 | 0.663/PDB |
| 306 | 44680 | 0.690/PDB | 640 | 22842 | 0.663/PDB |
| 312 | 250429 | 0.689/PDB | 648 | 83237 | 0.663/PDB |
| 313 | 250429 | 0.689/PDB | 670 | 159242 | 0.661/ChEMBL |
| 318 | 62129 | 0.689/PDB | 672 | 45527 | 0.661/ChEMBL |
| 323 | 16631 | 0.688/ChEMBL | 687 | 30205 | 0.661/PDB |
| 325 | 84200 | 0.688/PDB | 697 | 67546 | 0.660/PDB |

**Table S2C.** NCI molecules within the top 1% of the screened compound library for protein 1000006 Protein Tyrosine Phosphatase (PTP) *Homo sapiens*

| Rank | CAS or NSC # | Score/Source | Rank | CAS or NSC # | Score/Source |
| --- | --- | --- | --- | --- | --- |
| 11 | 325319 | 0.755/ChEMBL | 314 | 646976 | 0.654/ChEMBL |
| 45 | 335979 | 0.711/ChEMBL | 326 | 116397 | 0.653/ChEMBL |
| 49 | 16646 | 0.707/ChEMBL | 329 | 16722 | 0.653/ChEMBL |
| 80 | 7668 | 0.689/PDB | 347 | 110562 | 0.651/DrugBank |
| 94 | 120631 | 0.686/ChEMBL | 365 | 3223-07-2 | 0.650/ChEMBL |
| 95 | 8481 | 0.686/ChEMBL | 377 | 3391 | 0.649/ChEMBL |
| 103 | 50690 | 0.684/ChEMBL | 386 | 47619 | 0.648/PDB |
| 114 | 14540 | 0.682/PDB | 391 | 241998 | 0.648/PDB |
| 116 | 133002 | 0.681/ChEMBL | 398 | 35545 | 0.647/ChEMBL |
| 127 | 16437 | 0.678/ChEMBL | 410 | 50654 | 0.646/ChEMBL |
| 132 | 159686 | 0.678/ChEMBL | 413 | 122224 | 0.646/ChEMBL |
| 136 | 9064 | 0.677/PDB | 430 | 7950 | 0.645/ChEMBL |
| 160 | 16631 | 0.673/ChEMBL | 435 | 211787 | 0.644/ChEMBL |
| 167 | 156957 | 0.672/ChEMBL | 451 | 55-98-1 | 0.643/PDB |
| 169 | 22847 | 0.671/PDB | 473 | 408860 | 0.643/ChEMBL |
| 186 | 6731 | 0.668/ChEMBL | 474 | 172255 | 0.642/PDB |
| 190 | 5451-09-2 | 0.668/PDB | 484 | 50680 | 0.642/ChEMBL |
| 214 | 89759 | 0.665/ChEMBL | 496 | 16736 | 0.641/ChEMBL |
| 240 | 83237 | 0.661/PDB | 515 | 240502 | 0.640/PDB |
| 247 | 1614 | 0.661/PDB | 517 | 41066 | 0.640/ChEMBL |
| 269 | 62511 | 0.659/PDB | 529 | 122023 | 0.639/PDB |
| 272 | 133351 | 0.659/DrugBank | 537 | 3001 | 0.639/PDB |
| 276 | 7962 | 0.658/ChEMBL | 540 | 163802 | 0.638/ChEMBL |
| 282 | 92794 | 0.657/ChEMBL | 557 | 38352 | 0.638/PDB |
| 291 | 290311 | 0.657/PDB | 594 | 19970 | 0.635/ChEMBL |
| 297 | 50688 | 0.656/ChEMBL | 635 | 39047 | 0.634/ChEMBL |
| 301 | 409663 | 0.656/ChEMBL | 639 | 407628 | 0.634/PDB |
| 302 | 12865 | 0.656/ChEMBL | 641 | 164459 | 0.634/ChEMBL |
| 308 | 38743 | 0.655/PDB | 656 | 9168 | 0.633/PDB |
| 309 | 87352 | 0.654/PDB | 668 | 81463 | 0.632/ChEMBL |

**Table S2D.** NCI molecules within the top 1% of the screened compound library for tryptophanyl tRNA synthetase from *Homo sapiens*

| Rank | CAS or NSC # | Score/Source | Rank | CAS or NSC # | Score/Source |
| --- | --- | --- | --- | --- | --- |
| 2 | 152459-95-5 | 0.929/ChEMBL | 236 | 319012 | 0.735/ChEMBL |
| 3 | 557795-19-4 | 0.925/ChEMBL | 243 | 407628 | 0.733/PDB |
| 4 | 284461-73-0 | 0.925/ChEMBL | 247 | 8090 | 0.733/ChEMBL |
| 8 | 231277-92-2 | 0.924/ChEMBL | 257 | 9037 | 0.731/PDB |
| 10 | 863127-77-9 | 0.923/ChEMBL | 261 | 367480 | 0.731/ChEMBL |
| 11 | 635702-64-6 | 0.921/ChEMBL | 267 | 170621 | 0.730/PDB |
| 13 | 183319-69-9 | 0.920/ChEMBL | 285 | 3001 | 0.727/PDB |
| 14 | 226080 | 0.914/ChEMBL | 286 | 109086 | 0.727/ChEMBL |
| 15 | 53123-88-9 | 0.914/ChEMBL | 296 | 29874 | 0.726/PDB |
| 16 | 221019 | 0.913/ChEMBL | 325 | 304902 | 0.723/PDB |
| 17 | 221019 | 0.913/ChEMBL | 330 | 149647-78-9 | 0.722/ChEMBL |
| 19 | 159351-69-6 | 0.896/ChEMBL | 332 | 122131 | 0.722/ChEMBL |
| 22 | 19803 | 0.883/ChEMBL | 338 | 88882 | 0.722/ChEMBL |
| 23 | 75607-67-9 | 0.883/PDB | 343 | 240502 | 0.721/PDB |
| 24 | 72292 | 0.878/ChEMBL | 345 | 88795 | 0.720/ChEMBL |
| 31 | 35679 | 0.856/ChEMBL | 352 | 159686 | 0.720/ChEMBL |
| 33 | 63963 | 0.844/PDB | 353 | 49701 | 0.720/ChEMBL |
| 43 | 76988 | 0.825/PDB | 354 | 1751 | 0.720/PDB |
| 55 | 81703 | 0.803/PDB | 361 | 50648 | 0.719/ChEMBL |
| 58 | 184475-35-2 | 0.799/ChEMBL | 362 | 37168 | 0.719/ChEMBL |
| 62 | 335979 | 0.795/PDB | 377 | 55152 | 0.718/ChEMBL |
| 64 | 43512 | 0.794/PDB | 381 | 11141 | 0.717/PDB |
| 69 | 401077 | 0.791/PDB | 382 | 11668 | 0.717/ChEMBL |
| 73 | 173969 | 0.788/ChEMBL | 389 | 246415 | 0.716/ChEMBL |
| 76 | 241998 | 0.783/PDB | 390 | 123318-82-1 | 0.716/PDB |
| 79 | 121182 | 0.779/PDB | 400 | 102314 | 0.716/PDB |
| 81 | 73482 | 0.779/ChEMBL | 401 | 215276 | 0.715/PDB |
| 87 | 26744 | 0.775/PDB | 406 | 50690 | 0.715/ChEMBL |
| 91 | 22847 | 0.772/PDB | 410 | 7218 | 0.715/ChEMBL |
| 104 | 284200 | 0.768/PDB | 427 | 159031 | 0.714/ChEMBL |
| 109 | 34983 | 0.765/ChEMBL | 433 | 62511 | 0.713/PDB |
| 110 | 641571-10-0 | 0.765/ChEMBL | 449 | 51787 | 0.712/ChEMBL |
| 113 | 61888 | 0.764/PDB | 460 | 164464 | 0.711/ChEMBL |
| 114 | 15362 | 0.763/ChEMBL | 465 | 50680 | 0.711/ChEMBL |
| 117 | 375105 | 0.762/ChEMBL | 472 | 96911 | 0.711/ChEMBL |
| 122 | 71795 | 0.760/PDB | 479 | 42846 | 0.710/ChEMBL |
| 123 | 71795 | 0.760/PDB | 502 | 121032-29-9 | 0.709/PDB |
| 132 | 4291-63-8 | 0.756/PDB | 520 | 116508 | 0.708/ChEMBL |
| 134 | 280594 | 0.756/PDB | 534 | 27032 | 0.707/ChEMBL |
| 135 | 601359 | 0.756/ChEMBL | 542 | 34488 | 0.707/ChEMBL |
| 151 | 647136 | 0.749/ChEMBL | 547 | 105781 | 0.706/ChEMBL |
| 159 | 89720 | 0.748/PDB | 553 | 7668 | 0.706/PDB |
| 160 | 109813 | 0.748/ChEMBL | 566 | 145118 | 0.705/ChEMBL |
| 164 | 325319 | 0.747/PDB | 576 | 179818 | 0.705/ChEMBL |
| 174 | 25740 | 0.745/PDB | 592 | 10416 | 0.704/ChEMBL |
| 181 | 5426 | 0.743/PDB | 608 | 1614 | 0.703/PDB |
| 190 | 106506 | 0.742/PDB | 610 | 227309 | 0.702/ChEMBL |
| 191 | 9064 | 0.742/PDB | 627 | 37612 | 0.702/ChEMBL |
| 204 | 109174 | 0.739/ChEMBL | 638 | 1451 | 0.701/ChEMBL |
| 209 | 443913-73-3 | 0.738/ChEMBL | 643 | 63701 | 0.701/PDB |
| 210 | 3223-07-2 | 0.738/PDB | 661 | 27305 | 0.700/PDB |
| 214 | 37187 | 0.737/ChEMBL | 662 | 19115 | 0.700/ChEMBL |
| 219 | 112677 | 0.737/ChEMBL | 696 | 15776 | 0.699/ChEMBL |
| 231 | 27389 | 0.735/PDB |  |  |  |

**Table S2E.** NCI molecules within the top 1% of the screened compound library for the catalytic domain of cAMP-dependent kinase from *Homo sapiens.*

| Rank | CAS or NSC # | Score/Source | Rank | CAS or NSC # | Score/Source |
| --- | --- | --- | --- | --- | --- |
| 1 | 152459-95-5 | 0.927/PDB | 293 | 43409 | 0.681/PDB |
| 2 | 75607-67-9 | 0.882/PDB | 295 | 159031 | 0.681/PDB |
| 3 | 153330 | 0.874/PDB | 297 | 122111-03-9 | 0.681/PDB |
| 7 | 76988 | 0.845/PDB | 301 | 19803 | 0.680/PDB |
| 12 | 310113 | 0.798/PDB | 308 | 43271 | 0.680/DrugBank |
| 17 | 121182 | 0.784/PDB | 309 | 71866 | 0.679/DrugBank |
| 19 | 332670 | 0.780/DrugBank | 312 | 54645 | 0.679/PDB |
| 27 | 641571-10-0 | 0.764/PDB | 316 | 646976 | 0.679/PDB |
| 29 | 4291-63-8 | 0.761/PDB | 357 | 40306 | 0.675/PDB |
| 32 | 26744 | 0.760/PDB | 370 | 45153 | 0.674/PDB |
| 34 | 280594 | 0.757/PDB | 398 | 59-05-2 | 0.672/DrugBank |
| 35 | 62609 | 0.754/DrugBank | 399 | 125095 | 0.672/PDB |
| 39 | 25740 | 0.749/PDB | 406 | 19063 | 0.671/PDB |
| 45 | 275266 | 0.744/PDB | 413 | 69-74-9 | 0.671/PDB |
| 56 | 123318-82-1 | 0.737/PDB | 414 | 23248 | 0.671/DrugBank |
| 59 | 5426 | 0.734/PDB | 416 | 345845 | 0.671/PDB |
| 69 | 70895 | 0.729/PDB | 435 | 14767 | 0.670/PDB |
| 76 | 9037 | 0.723/PDB | 448 | 128737 | 0.669/DrugBank |
| 87 | 38845 | 0.718/PDB | 451 | 81703 | 0.669/PDB |
| 104 | 121032-29-9 | 0.710/PDB | 463 | 154361-50-9 | 0.668/PDB |
| 105 | 61610 | 0.710/DrugBank | 497 | 36586 | 0.666/PDB |
| 107 | 128751 | 0.709/DrugBank | 510 | 268251 | 0.666/PDB |
| 117 | 63701 | 0.707/PDB | 517 | 109719 | 0.665/PDB |
| 128 | 27305 | 0.705/PDB | 527 | 71795 | 0.665/PDB |
| 150 | 42135 | 0.700/DrugBank | 528 | 71795 | 0.665/PDB |
| 158 | 324623 | 0.699/PDB | 531 | 8519 | 0.664/PDB |
| 185 | 61642 | 0.695/PDB | 533 | 46615 | 0.664/PDB |
| 197 | 38007 | 0.693/PDB | 535 | 60659 | 0.664/PDB |
| 205 | 77913 | 0.693/PDB | 556 | 46385 | 0.663/PDB |
| 218 | 27032 | 0.691/PDB | 559 | 50-91-9 | 0.663/PDB |
| 219 | 147829 | 0.690/PDB | 578 | 62129 | 0.662/DrugBank |
| 232 | 51349 | 0.688/PDB | 595 | 15776 | 0.661/PDB |
| 233 | 105827 | 0.688/PDB | 622 | 9852 | 0.660/PDB |
| 234 | 318799 | 0.688/PDB | 628 | 22801 | 0.660/PDB |
| 242 | 109174 | 0.687/PDB | 633 | 57890 | 0.660/DrugBank |
| 247 | 79010 | 0.687/PDB | 660 | 99657 | 0.658/PDB |
| 248 | 39984 | 0.687/PDB | 669 | 97104 | 0.658/DrugBank |
| 260 | 49701 | 0.685/DrugBank | 676 | 122131 | 0.658/PDB |
| 286 | 154718 | 0.682/PDB | 677 | 227309 | 0.658/PDB |
| 288 | 29200 | 0.682/DrugBank | 691 | 87010 | 0.657/PDB |
| 292 | 106570 | 0.681/PDB | 692 | 116644 | 0.657/PDB |

**Table S2F.** NCI molecules within the top 1% of the screened compound library for thioredoxin peroxidase 2 from *Plasmodium falciparum*

| Rank | CAS or NSC # | Score/Source | Rank | CAS or NSC # | Score/Source |
| --- | --- | --- | --- | --- | --- |
| 14 | 284200 | 0.822/PDB | 332 | 240502 | 0.712/ChEMBL |
| 28 | 151252 | 0.792/PDB | 344 | 26349 | 0.711/ChEMBL |
| 36 | 102314 | 0.783/PDB | 381 | 524615 | 0.708/ChEMBL |
| 59 | 16416 | 0.764/ChEMBL | 383 | 407628 | 0.708/ChEMBL |
| 65 | 106506 | 0.760/ChEMBL | 391 | 7962 | 0.707/ChEMBL |
| 66 | 62511 | 0.759/ChEMBL | 403 | 16722 | 0.706/ChEMBL |
| 69 | 22847 | 0.756/ChEMBL | 415 | 57345 | 0.705/ChEMBL |
| 74 | 16631 | 0.754/PDB | 438 | 2561 | 0.703/ChEMBL |
| 75 | 101758 | 0.754/PDB | 450 | 57608 | 0.701/ChEMBL |
| 78 | 335979 | 0.752/ChEMBL | 461 | 55172 | 0.701/ChEMBL |
| 97 | 9064 | 0.748/PDB | 462 | 89759 | 0.701/ChEMBL |
| 120 | 241998 | 0.741/ChEMBL | 495 | 11141 | 0.699/ChEMBL |
| 138 | 78846 | 0.737/ChEMBL | 501 | 38743 | 0.698/ChEMBL |
| 139 | 156957 | 0.737/ChEMBL | 503 | 50-35-1 | 0.698/ChEMBL |
| 162 | 3001 | 0.733/PDB | 529 | 250429 | 0.696/ChEMBL |
| 166 | 66020 | 0.732/ChEMBL | 530 | 250429 | 0.696/ChEMBL |
| 169 | 1847 | 0.731/DrugBank | 540 | 121781 | 0.695/ChEMBL |
| 179 | 373535 | 0.729/ChEMBL | 543 | 40614 | 0.695/ChEMBL |
| 186 | 106231 | 0.728/ChEMBL | 557 | 120631 | 0.695/PDB |
| 189 | 325319 | 0.728/ChEMBL | 584 | 122376 | 0.693/ChEMBL |
| 210 | 29874 | 0.726/ChEMBL | 587 | 22881 | 0.693/ChEMBL |
| 219 | 16646 | 0.725/PDB | 597 | 215276 | 0.692/ChEMBL |
| 226 | 25368 | 0.724/PDB | 615 | 168027 | 0.691/ChEMBL |
| 243 | 8481 | 0.723/ChEMBL | 621 | 241624 | 0.691/ChEMBL |
| 269 | 6101 | 0.718/ChEMBL | 626 | 16736 | 0.691/ChEMBL |
| 288 | 157725 | 0.716/ChEMBL | 636 | 5451-09-2 | 0.690/PDB |
| 292 | 20192 | 0.715/PDB | 640 | 7436 | 0.689/ChEMBL |
| 295 | 7668 | 0.715/PDB | 649 | 97538 | 0.689/PDB |
| 299 | 290311 | 0.715/ChEMBL | 654 | 244387 | 0.689/ChEMBL |
| 307 | 122224 | 0.714/ChEMBL | 655 | 38352 | 0.689/ChEMBL |
| 308 | 284437 | 0.714/ChEMBL | 666 | 2952 | 0.688/ChEMBL |
| 314 | 5476 | 0.713/ChEMBL | 687 | 330753 | 0.687/ChEMBL |
| 321 | 105584 | 0.713/ChEMBL | 689 | 81703 | 0.687/ChEMBL |
| 329 | 1614 | 0.712/ChEMBL | 694 | 109128 | 0.686/ChEMBL |

**Table S2G.** NCI molecules within the top 1% of the screened compound library for ubiquitin conjugating enzyme from *Plasmodium falciparum*

| Rank | CAS or NSC # | Score/Source | Rank | CAS or NSC # | Score/Source |
| --- | --- | --- | --- | --- | --- |
| 2 | 152459-95-5 | 0.932/ChEMBL | 374 | 351691 | 0.679/ChEMBL |
| 3 | 284461-73-0 | 0.928/ChEMBL | 382 | 131467 | 0.679/ChEMBL |
| 4 | 557795-19-4 | 0.927/ChEMBL | 394 | 240502 | 0.677/DrugBank |
| 6 | 231277-92-2 | 0.926/ChEMBL | 399 | 319029 | 0.677/ChEMBL |
| 7 | 863127-77-9 | 0.925/ChEMBL | 404 | 134577 | 0.677/ChEMBL |
| 9 | 635702-64-6 | 0.921/ChEMBL | 423 | 112975 | 0.676/ChEMBL |
| 10 | 183319-69-9 | 0.921/ChEMBL | 428 | 343557 | 0.675/ChEMBL |
| 16 | 184475-35-2 | 0.800/ChEMBL | 430 | 50651 | 0.675/ChEMBL |
| 30 | 641571-10-0 | 0.768/ChEMBL | 435 | 204232 | 0.674/ChEMBL |
| 31 | 34983 | 0.767/ChEMBL | 439 | 124146 | 0.674/ChEMBL |
| 45 | 241998 | 0.751/DrugBank | 441 | 201863 | 0.674/ChEMBL |
| 69 | 367480 | 0.733/ChEMBL | 454 | 17129 | 0.673/ChEMBL |
| 96 | 149647-78-9 | 0.720/ChEMBL | 470 | 25678 | 0.672/ChEMBL |
| 98 | 88795 | 0.718/ChEMBL | 471 | 165883 | 0.672/ChEMBL |
| 101 | 22847 | 0.715/DrugBank | 473 | 42135 | 0.672/ChEMBL |
| 105 | 49701 | 0.713/ChEMBL | 495 | 13248 | 0.670/ChEMBL |
| 113 | 87010 | 0.712/ChEMBL | 513 | 40306 | 0.669/ChEMBL |
| 125 | 116508 | 0.710/ChEMBL | 516 | 35545 | 0.669/ChEMBL |
| 128 | 443913-73-3 | 0.709/ChEMBL | 521 | 77913 | 0.669/ChEMBL |
| 143 | 34488 | 0.706/ChEMBL | 531 | 8090 | 0.669/ChEMBL |
| 160 | 37612 | 0.703/ChEMBL | 534 | 43271 | 0.669/ChEMBL |
| 176 | 1451 | 0.701/ChEMBL | 536 | 50654 | 0.669/ChEMBL |
| 183 | 366802 | 0.699/ChEMBL | 540 | 76549 | 0.668/ChEMBL |
| 184 | 308835 | 0.699/ChEMBL | 551 | 71866 | 0.668/ChEMBL |
| 188 | 319012 | 0.699/ChEMBL | 566 | 143241 | 0.667/ChEMBL |
| 196 | 55152 | 0.698/ChEMBL | 568 | 293962 | 0.667/ChEMBL |
| 202 | 366-70-1 | 0.697/ChEMBL | 569 | 11667 | 0.667/ChEMBL |
| 205 | 7218 | 0.697/ChEMBL | 573 | 373535 | 0.667/ChEMBL |
| 235 | 150114 | 0.692/ChEMBL | 582 | 158959 | 0.666/ChEMBL |
| 243 | 20045 | 0.691/ChEMBL | 590 | 33010 | 0.665/ChEMBL |
| 256 | 637578 | 0.689/ChEMBL | 591 | 38352 | 0.665/ChEMBL |
| 270 | 15362 | 0.688/ChEMBL | 614 | 6866 | 0.664/ChEMBL |
| 296 | 93427 | 0.685/ChEMBL | 634 | 14311 | 0.664/ChEMBL |
| 304 | 366801 | 0.684/ChEMBL | 652 | 19061 | 0.663/ChEMBL |
| 307 | 36582 | 0.684/ChEMBL | 653 | 11668 | 0.663/ChEMBL |
| 314 | 33005 | 0.684/ChEMBL | 654 | 60387 | 0.663/ChEMBL |
| 330 | 59776 | 0.683/ChEMBL | 680 | 653004 | 0.662/ChEMBL |
| 350 | 241624 | 0.681/ChEMBL | 684 | 12865 | 0.662/ChEMBL |
| 355 | 127133 | 0.681/ChEMBL | 686 | 106570 | 0.662/ChEMBL |
| 363 | 122224 | 0.680/DrugBank | 693 | 106506 | 0.662/ChEMBL |

**Table S2H.** NCI molecules within the top 1% of the screened compound library for nucleosome assembly protein 1 from *Plasmodium knowlesi*

| Rank | CAS or NSC # | Score/Source | Rank | CAS or NSC # | Score/Source |
| --- | --- | --- | --- | --- | --- |
| 1 | 152459-95-5 | 0.941/ChEMBL | 300 | 141538 | 0.710/PDB |
| 5 | 407286 | 0.915/ChEMBL | 306 | 17128 | 0.709/PDB |
| 11 | 614552 | 0.865/PDB | 312 | 21603 | 0.709/PDB |
| 15 | 121182 | 0.839/PDB | 323 | 301683 | 0.708/ChEMBL |
| 20 | 107022 | 0.827/ChEMBL | 334 | 5451-09-2 | 0.706/PDB |
| 22 | 284200 | 0.818/PDB | 335 | 179822 | 0.706/ChEMBL |
| 30 | 4291-63-8 | 0.810/PDB | 336 | 15780 | 0.705/PDB |
| 35 | 250429 | 0.802/PDB | 346 | 57345 | 0.705/PDB |
| 36 | 250429 | 0.802/PDB | 350 | 105827 | 0.704/PDB |
| 41 | 25740 | 0.797/PDB | 377 | 81660 | 0.700/PDB |
| 44 | 1751 | 0.792/PDB | 390 | 29874 | 0.699/PDB |
| 48 | 129536 | 0.788/PDB | 392 | 84200 | 0.699/PDB |
| 49 | 129536 | 0.788/PDB | 399 | 69-74-9 | 0.697/PDB |
| 50 | 75607-67-9 | 0.785/PDB | 400 | 45117 | 0.697/PDB |
| 53 | 102314 | 0.781/PDB | 404 | 70895 | 0.697/PDB |
| 55 | 22842 | 0.780/ChEMBL | 409 | 86467 | 0.696/PDB |
| 71 | 25368 | 0.770/PDB | 429 | 49643 | 0.694/PDB |
| 72 | 641571-10-0 | 0.770/ChEMBL | 435 | 122111-03-9 | 0.693/PDB |
| 74 | 244387 | 0.769/PDB | 446 | 87010 | 0.692/ChEMBL |
| 76 | 209870 | 0.766/PDB | 447 | 59620 | 0.692/PDB |
| 82 | 47619 | 0.762/PDB | 448 | 263164 | 0.692/PDB |
| 87 | 123318-82-1 | 0.759/PDB | 453 | 168027 | 0.691/PDB |
| 88 | 101758 | 0.758/PDB | 464 | 345647 | 0.690/PDB |
| 101 | 44680 | 0.754/PDB | 465 | 345647 | 0.690/PDB |
| 107 | 9064 | 0.752/PDB | 467 | 47617 | 0.690/PDB |
| 115 | 8481 | 0.749/PDB | 477 | 34875 | 0.689/PDB |
| 116 | 121032-29-9 | 0.749/PDB | 496 | 120631 | 0.687/PDB |
| 145 | 196515 | 0.740/PDB | 523 | 35611 | 0.684/PDB |
| 147 | 62511 | 0.740/PDB | 529 | 144982 | 0.684/PDB |
| 152 | 27305 | 0.737/PDB | 536 | 217306 | 0.683/PDB |
| 158 | 3001 | 0.736/PDB | 546 | 36398 | 0.682/PDB |
| 187 | 45923 | 0.729/ChEMBL | 558 | 9665 | 0.681/PDB |
| 188 | 298-81-7 | 0.729/ChEMBL | 564 | 110899 | 0.681/ChEMBL |
| 195 | 63701 | 0.727/PDB | 572 | 55172 | 0.680/PDB |
| 215 | 2561 | 0.723/PDB | 589 | 14975 | 0.679/PDB |
| 218 | 37219 | 0.723/PDB | 592 | 290311 | 0.679/PDB |
| 234 | 43409 | 0.721/PDB | 608 | 38983 | 0.677/PDB |
| 238 | 20192 | 0.719/PDB | 616 | 227309 | 0.677/PDB |
| 251 | 7668 | 0.717/PDB | 628 | 400978 | 0.676/PDB |
| 274 | 318799 | 0.713/PDB | 633 | 117446 | 0.676/PDB |
| 284 | 97538 | 0.711/PDB | 651 | 62840 | 0.674/PDB |
| 286 | 45153 | 0.711/PDB | 660 | 157035 | 0.673/PDB |
| 287 | 1614 | 0.711/PDB | 675 | 280594 | 0.672/PDB |

**Table S3**. Summary of virtual ligand screening, thermal shift assay, binding parameters and antibacterial, antifungal and anticancer properties for the hits obtained on *E. coli* DHFR.

| Identity (NSC) | Rank^a^ | TC^b^ | Q^#^ | Δ*T*_m_ | K_D_ (nM)^c^  (Approx) | DH5α  (MIC) | MDREC  (MIC) | MRSA  (MIC) | VREF  (MIC) | HCT-116  (IC-50) |
| --- | --- | --- | --- | --- | --- | --- | --- | --- | --- | --- |
| 309401 | 253/52 | 0.675753 | 0.5 | 30.74 | 48.21 | 7.813 | 125 | 31.25 | 31.25 | 0.130 |
| 740* | 2/1 | 0.934113 | 0.5 | 29.77 | 62.25 | ND | ND | ND | 500 | 0.048 |
| 339578* | 317/58 | 0.669547 | 1.4 | 27.57 | 114.61 | 62.5 | 250 | 31.25 | 31.25 | 6.11 |
| 382035* | 46/13 | 0.731313 | 0.5 | 24.58 | 266.13 | ND | ND | 31.25 | 31.25 | 0.182 |
| 754230* | 27/10 | 0.752937 | 0.1 | 22.38 | 499.42 | ND | ND | ND | ND | <<0.031 |
| 698037* | 3/2 | 0.930956 | 1.3 | 21.88 | 576.90 | ND | ND | ND | 500 | ND |
| 80735 | 658/98 | 0.648163 | 0.3 | 18.16 | 1710.28 | ND | ND | ND | ND | 10.9 |
| 61642* | 197/41 | 0.683773 | 0.6 | 17.58 | 2030.55 | ND | ND | ND | ND | ND |
| 123458 | 644/97 | 0.648675 | 0.6 | 14.17 | 5639.99 | ND | ND | ND | ND | ND |
| 159686 | 684/103 | 0.646950 | 0.6 | 11.52 | 12662.83 | ND | ND | ND | ND | ND |
| 157522 | 351/64 | 0.666258 | 0.4 | 10.13 | 19457.54 | ND | ND | ND | ND | ND |
| 379536 | 628/95 | 0.649246 | 0.3 | 8.86 | 28903.82 | ND | ND | ND | ND | ND |
| 55152 | 661/99 | 0.648082 | 0.2 | 5.89 | 73833.22 | ND | ND | ND | ND | ND |
| 130801 | 181/38 | 0.686146 | 1.1 | 5.84 | 75019.46 | ND | ND | ND | ND | ND |
| 50690 | 547/86 | 0.653097 | 0.3 | 0.23 | 463384.94 | ND | ND | ND | ND | ND |

* Indicates reported inhibitors of DHFR independently picked up by our predictions and validated experimentally. ^a^The rank is indicated as total rank across the 69,683 compounds including ZINC background/rank specified only for the NCI set of 1812 compounds. ^b^ Tanimoto coefficient, #, quality score (Q) is the ratio of melting-associated increase in fluorescence (ΔF_melt_) and total range in fluorescence (ΔF_total_). A Q value of 1 represents a high-quality curve, while a value of 0 shows an absence of melting as described earlier (Crowther *et al.,* 2010). K_D_: see Table 4 footnotes. MIC, IC-50, ND, DH5α, MRSA, MDREC, VREF, HCT-116: See Table 5 footnotes. ^c^ The dissociation constants reported are in this table are computed from the thermal shifts obtained.

**Table S4.** Summary of virtual ligand screening, thermal shift assay, binding parameters and anticancer properties for the hits obtained on protein tyrosine phosphatases from *R. norvegicus* and *H. sapiens* and tryptophanyl tRNA synthetase from *H. sapiens*.

| Protein | Ligand (NSC) | Rank^a^ | TC^b^ | Q^#^ | Δ*T*_m_ | K_D_(nM)^c^  (Approx) | HCT-116 (IC-50) |
| --- | --- | --- | --- | --- | --- | --- | --- |
| **1000001**^1^ | 134137 | 201/29 | 0.707704 | 0.01 | 12.3 | 406.04 | ND |
|  | 45527 | 672/88 | 0.661702 | 0.04 | 11.7 | 567.24 | ND |
|  | 111552 | 112/19 | 0.725407 | 0.007 | 11.3 | 709.37 | 2.2 |
|  | 156563 | 102/17 | 0.729834 | 0.004 | 10.2 | 1315.78 | ND |
|  | 246131^@^ | 528/75 | 0.670487 | 0.2 | 9.99 | 1472.88 | 0.024 |
|  | 88882 | 40/9 | 0.760550 | 0.8 | 9.98 | 1472.88 | 4.44 |
|  | 153172 | 412/56 | 0.680384 | 0.1 | 9.3 | 2188.28 | ND |
|  | 106863 | 227/32 | 0.704244 | 1.1 | 7.4 | 6466.21 | 14.5 |
|  | 30205 | 687/89 | 0.661070 | 0.01 | 7.3 | 6848.16 | 0.146 |
|  | 1847 | 193/27 | 0.709478 | 0.5 | 4.1 | 43813.93 | ND |
| **1000006**^2^ | 133351 | 272/22 | 0.659134 | 0.1 | 16.76 | 168.29 | ND |
|  | 16736 | 496/48 | 0.641747 | 0.4 | 4.89 | 44478.35 | ND |
|  | 50690 | 103/7 | 0.684946 | 0.8 | 4.59 | 51486.30 | ND |
|  | 16722 | 329/33 | 0.653290 | 0.4 | 3.09 | 107448.90 | ND |
|  | 92794 | 282/24 | 0.657786 | 0.004 | 2.26 | 161917.65 | 9.78 |
|  | 7950 | 430/42 | 0.645329 | 0.3 | 1.22 | 271496.12 | ND |
| **TrpRS**^3^ | 750690^¥^ | 3/2 | 0.925710 | 1.3 | 14.57 | 1277.51 | 1.11 |
|  | 88882 | 338/67 | 0.722035 | 0.6 | 12.07 | 3827.52 | 4.44 |
|  | 50690 | 406/82 | 0.715421 | 0.8 | 10.27 | 7738.83 | ND |
|  | 37168 | 362/74 | 0.719466 | 0.4 | 9.9 | 9058.88 | 1.34 |
|  | 55152 | 377/75 | 0.718194 | 0.95 | 6.17 | 39602.03 | ND |

^1, 2, 3^ See Table 4 footnotes; ND, IC-50, HCT-116, ^@, ¥^  see Table 5 footnotes. ^a^, ^b^,^c^ ,^#^, see Table S3 footnotes.

**Table S5**. Summary of virtual ligand screening, thermal shift assay and binding parameters for the hits obtained on *P. falciparum* Ubiquitin conjugating enzyme, Human wild-type cAMP-dependent protein kinase catalytic subunit, *P. knowlesi* nucleosome assembly protein 1 and *P. falciparum* Thioredoxin peroxidase 2.

| Proteins* | Ligand Identity(NSC) | Rank^a^ | TC^b^ | Q^#^ | Δ*T*_m_ | *K*_D_ (μM)^c^  (Approx) |
| --- | --- | --- | --- | --- | --- | --- |
| *P. falciparum* UCE | 93427 | 296/33 | 0.685470 | 0.05 | 14.86 | 1.38 |
|  | 50651 | 430/48 | 0.675238 | 0.13 | 2.25 | 196.73 |
| Human cDPK | 27032 | 218/30 | 0.691233 | 1.0 | 2.95 | 48.54 |
|  | 97104 | 669/78 | 0.658402 | 0.5 | 1.01 | 200.24 |
|  | 61642 | 185/27 | 0.695098 | 0.4 | 0.71 | 275.20 |
| *P. knowlesi*  NAP1 | 36398 | 546/74 | 0.682738 | 0.2 | 2.21 | 180.14 |
|  | 63878 | 399/56 | 0.697887 | 1.1 | 2.18 | 182.63 |
|  | 34875 | 477/69 | 0.689870 | 0.6 | 1.74 | 223.55 |
|  | 227309 | 616/81 | 0.677021 | 0.8 | 0.64 | 371.49 |
| *P. falciparum* TP2 | 106231 | 186/19 | 0.728830 | 0.54 | 5.7 | 40.87 |
|  | 16736 | 626/59 | 0.691186 | 0.21 | 3.8 | 122.61 |

^a^ , ^b^,^c^, ^#^ see Table S3 footnotes

**Table S6.** Comparative parameters for known binders of 1000001 tested on 1000006

| S. No. | Small mol. (NSC) | 1000001^¶^ | | 1000006^¶^ | |
| --- | --- | --- | --- | --- | --- |
|  |  | Δ*T*_m_ | Rank^a^ | Δ*T*_m_ | Rank^a^ |
| 1 | 88882 | 9.79 | 40/9 | 4.39 | 16171/794 |
| 2 | 156563 | 10.66 | 102/17 | 5.36 | 2326/192 |
| 3 | 111552 | 37.24 | 112/19 | 29.52 | 1092/96 |
| 4 | 1847 | 4.23 | 193/27 | 0 | 3951/292 |
| 5 | 134137 | 11.33 | 201/29 | 1.36 | 9872/551 |
| 6 | 106863 | 7.34 | 227/32 | 0 | 2114/173 |
| 7 | 153172 | 9.70 | 412/56 | 5.48 | 1300/116 |
| 8 | 246131 | 10.31 | 528/75 | 5.62 | 6184/391 |
| 9 | 45527 | 11.79 | 672/88 | 5.48 | 16437/802 |
| 10 | 30205 | 9.91 | 687/89 | Ambiguous | 6950/424 |

^¶^ See Table 4 footnotes. ^a^see Table S3 footnotes. Text in bold indicates those molecules that either yielded non-interpretable 2 step unfolding curves or those that didn’t show a significant thermal shift.

**Fig S1.** Standardization of the thermal melt assay. **A)** Thermal melt transition of citrate synthase, malate dehydrogenase and carbonic anhydrase, **B)** Thermal melt assay of carbonic anhydrase (CA) at several fixed concentrations of trifluoromethane- sulfonamide (TFMSA), **C)** Thermal melt assay of citrate synthase (CS) at several fixed concentrations of oxaloacetate (OA), **D)** Thermal melt assay of malate dehydrogenase (MD) at several fixed concentrations of nicotinamide adenine dinucleotide (NADH). Inset shows the first derivative transformation of the respective curves in each plot.

**
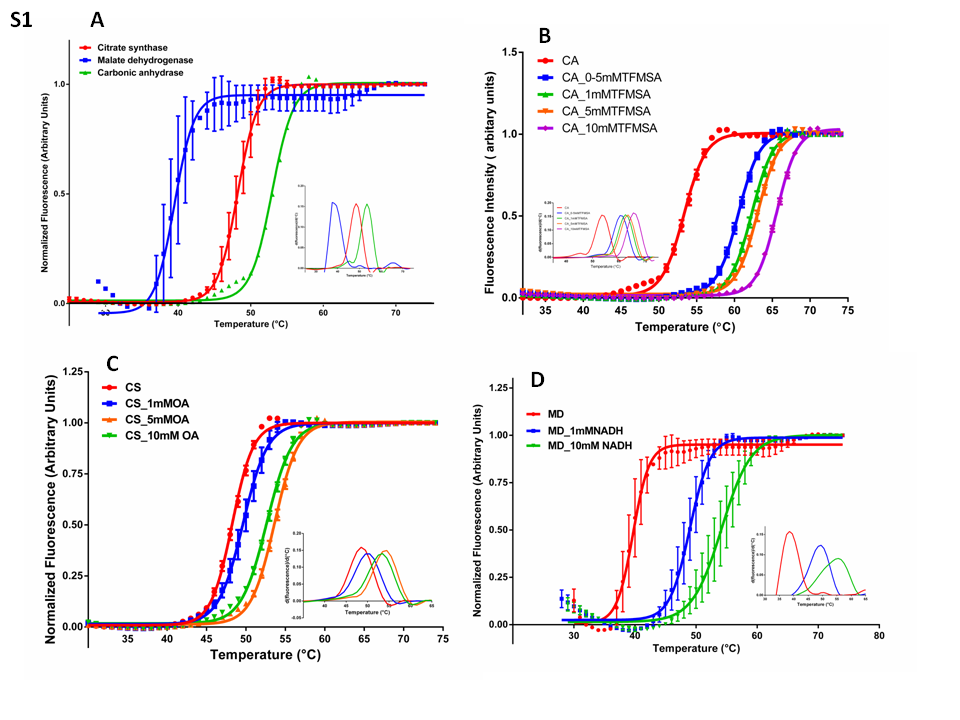
**

**Fig S2**. Grouping of experimental hits for various proteins into distinct clusters based on their identity assessed by a Tanimoto coefficient cut-off of 0.7 **A)** Dihydrofolate reductase **B)** PTP 1000001 **C)** PTP 1000006 D) Tryptophanyl tRNA synthetase. Boxed regions represent the various clusters with the identity of the small-molecules specified by their NSC number.


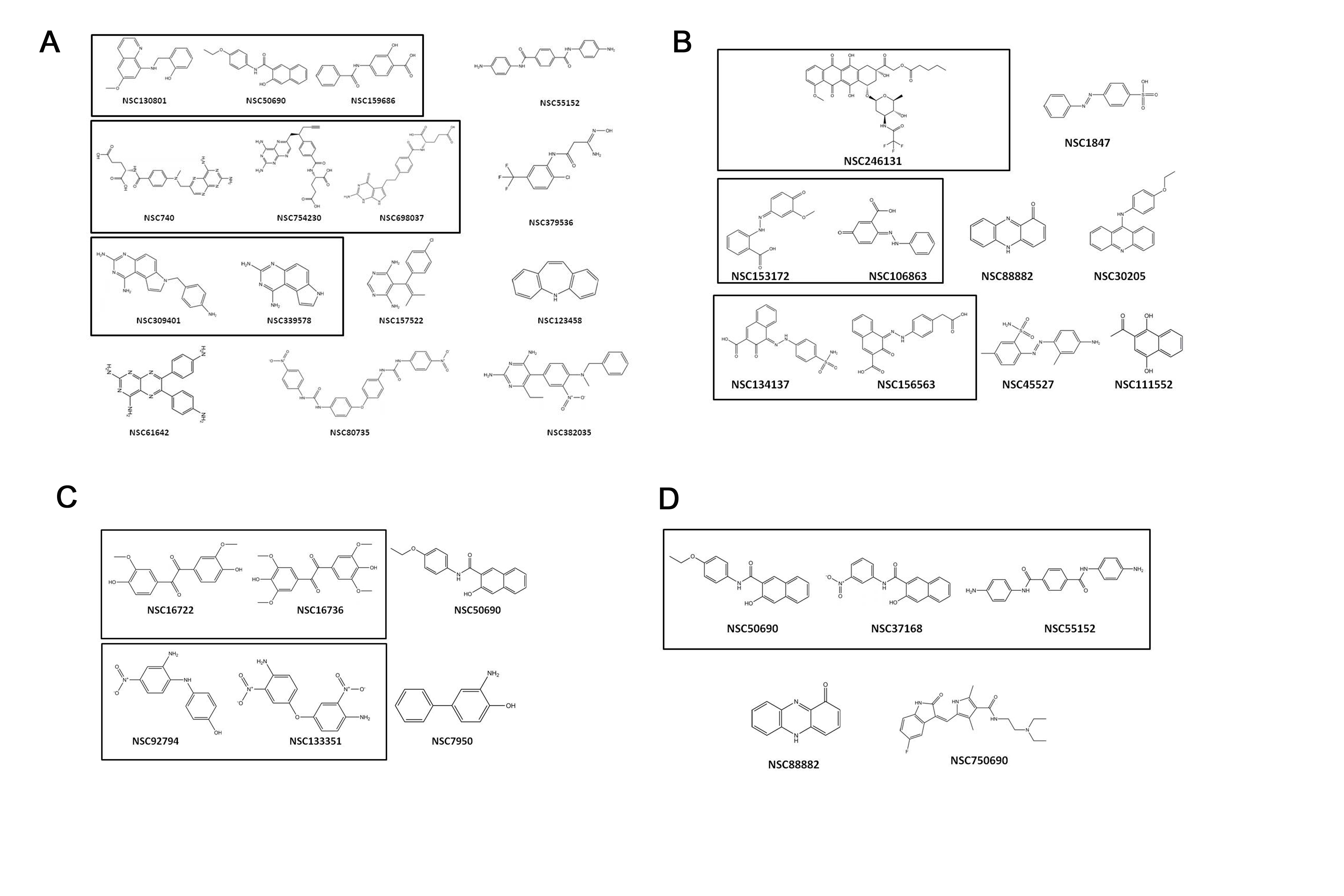

Supplement: Additional file 1 — Detailed FINDSITEcomb VLS results, Thermal shift assay standardization: methods and results, HTS protocol table, detailed results on the thermal shift assay and biological activity assay for the eight protein in tabular form, discussion on the differences between 1000001 and 1000006 VLS and experimental overlap and figure depicting the diversity of compounds picked up by the current methodology. [file 1758-2946-6-16-S1.docx]
